# Resolving the structure of phage–bacteria interactions in the context of natural diversity

**DOI:** 10.1038/s41467-021-27583-z

**Published:** 2022-01-18

**Authors:** Kathryn M. Kauffman, William K. Chang, Julia M. Brown, Fatima A. Hussain, Joy Yang, Martin F. Polz, Libusha Kelly

**Affiliations:** 1 Department of Civil and Environmental Engineering, Massachusetts Institute of Technology, Cambridge, MA 02139 USA; 2 Department of Systems and Computational Biology, Albert Einstein College of Medicine, Bronx, NY 10461 USA; 3 Division of Microbial Ecology, Department of Microbiology and Ecosystem Science, Centre for Microbiology and Environmental Systems Science, University of Vienna, Vienna, Austria; 4 Department of Microbiology and Immunology, Albert Einstein College of Medicine, Bronx, NY 10461 USA; 5 Present Address: Department of Oral Biology, The University at Buffalo, Buffalo, NY 14214 USA; 6 Present Address: Bigelow Laboratory for Ocean Sciences, East Boothbay, ME 04544 USA; 7 Present Address: Ragon Institute of MGH, MIT, and Harvard, Cambridge, MA 02139 USA

**Keywords:** Microbial ecology, Bacteriophages, Marine biology

## Abstract

Microbial communities are shaped by viral predators. Yet, resolving which viruses (phages) and bacteria are interacting is a major challenge in the context of natural levels of microbial diversity. Thus, fundamental features of how phage-bacteria interactions are structured and evolve in the wild remain poorly resolved. Here we use large-scale isolation of environmental marine *Vibrio* bacteria and their phages to obtain estimates of strain-level phage predator loads, and use all-by-all host range assays to discover how phage and host genomic diversity shape interactions. We show that lytic interactions in environmental interaction networks (as observed in agar overlay) are sparse—with phage predator loads being low for most bacterial strains, and phages being host-strain-specific. Paradoxically, we also find that although overlap in killing is generally rare between tailed phages, recombination is common. Together, these results suggest that recombination during cryptic co-infections is an important mode of phage evolution in microbial communities. In the development of phages for bioengineering and therapeutics it is important to consider that nucleic acids of introduced phages may spread into local phage populations through recombination, and that the likelihood of transfer is not predictable based on lytic host range.

## Abstract (web summary)

Understanding the interactions between bacteria and their viruses (phages) in natural communities is a major challenge. Here, the authors isolate and study large numbers of marine *Vibrio* bacteria and their phages, and find that lytic interactions are sparse and many phages are host-strain-specific, but nevertheless recombination between some phages is common.

## Introduction

Phages are important predators of bacteria—they shape the structure, function, and evolution of natural microbial communities, and they are potential tools to manipulate microbial communities for industrial, bioengineering, and therapeutic applications^1^. Key to understanding the roles of phages in natural communities, and to their design and use as efficient and robust tools, is knowledge of their host ranges in the context of the systems in which they exist or will be used^2^. Yet, how phage host ranges are structured in complex microbial communities remains challenging to address^3^ because the local genomic diversity of phage and bacterial strains is high, and phage-bacteria interactions are specific. Thus, resolving the structure of interactions at the strain-level requires systematic assays of host ranges of phages against panels of potential host strains. The largest such study in the context of natural microbial communities was performed by Moebus and Nattkemper in the 1970s^4^. Later re-analyses of the structure of the Moebus-Nattkemper matrix by Flores et al. in 2013^5^ found this network to have a statistically modular structure and numerous singleton interactions. This confirmed predictions made by Flores et al., in their prior large scale meta-analysis of 38 phage-bacteria interaction networks (PBINs)^6^, that whereas interactions in laboratory PBINs were largely nested, larger environmental sampling would reveal modularity in interactions. However, as neither phages nor bacteria of the Moebus-Nattkemper matrix were genome sequenced, the relation of the observed modules to bacterial and phage genomic diversity could not be determined – and thus how phage and bacterial phylogenetic diversity shape the structure of PBINs in natural communities remains unclear.

In this work, we analyze a PBIN for which genomes of the majority of member phages and bacteria have been sequenced to address how environmental PBINs are structured in marine microbial communities. We show that the biological basis of modular structure in large-scale PBINs varies across modules and can be defined by either phage or bacterial phylogenetic boundaries; and we find that whereas overlaps in killing host ranges of phages are rare, local pools of phage genomes are highly recombined. We propose two models that reconcile the contrasting low overlap in killing among tailed phages with the prevalence of recombined genomes, and point to cryptic co-infections of bacteria by multiple phages as being important in the ecology and evolution of phage-bacteria interactions in microbial communities.

## Results

### Co-occurring lytic phage predator loads appear low

To evaluate phage predation on closely related bacteria in the environment, we focused on the well-characterized^7–10^ coastal marine heterotrophic *Vibrionaceae* bacteria as a model system. We isolated 1440 strains, predominantly in the genus *Vibrio*, over three days (ordinal day 222, 261, and 286) during the course of the 3-month 2010 Nahant Collection Time Series^11^ and sequenced the housekeeping gene *hsp60* to initially resolve their phylogenetic relationships. Using these isolates as bait we quantified concentrations of lytic phages present for each strain in seawater collected on the same days. By using direct plating agar overlay methods^12,13^ with virus concentrates^14^, rather than enrichments, we were able to obtain estimates of concentrations of co-occurring plaque-forming phages for each bacterial strain. In previous work^14^ we showed that use of oxalate solution in this viral concentration procedure allows initial recovery of 49–55% of infective viruses (see Methods), as well as stable storage - thus, direct and doubled counts provide approximate lower and upper bounds of plaque forming units (PFUs) per ml of seawater concentrate in these assays. Of the 1287 total bacterial strains which both grew for the bait assay and for which we were able to obtain *hsp60* gene sequences, 285 (22%) were plaque positive – revealing sensitivity to killing by co-occurring phages.

**Fig. 1:**
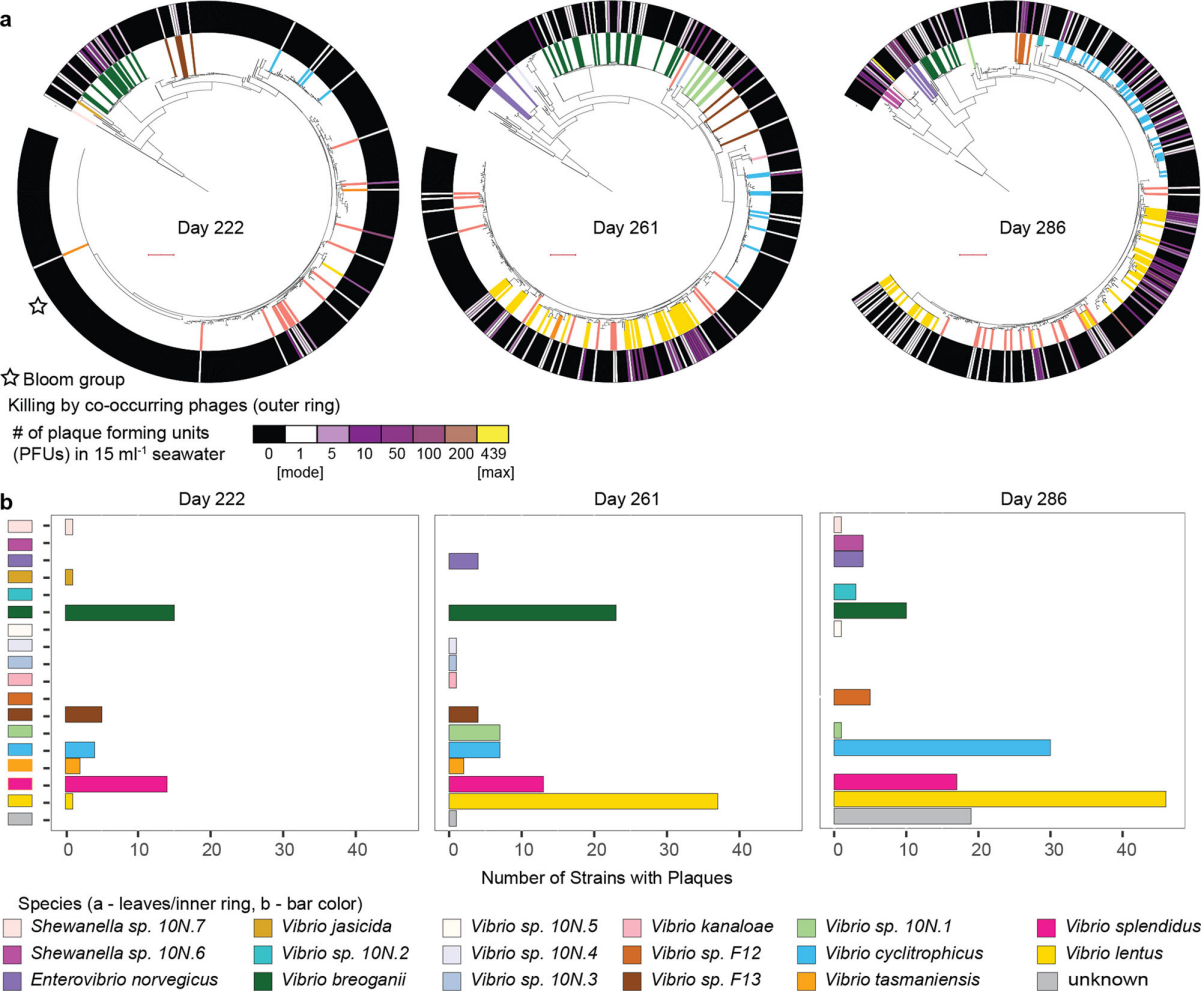
Phage predation pressure on individual bacterial strains appears low overall, is not uniform among closely related bacterial isolates, and varies across days of sampling. **a** Phylogenetic relationships between *Vibrionaceae* strains isolated on each of three days and screened for sensitivity to phages in seawater concentrates from the same days (449 bacterial isolates on ordinal day 222; 443 on day 261; 395 on day 286; shown as *hsp60* gene trees with leaves colored by host species for isolates with sequenced genomes). Sensitivity to phage killing is shown in the outer ring, with colors representing the number of plaques formed on each strain. The majority of bacterial isolates screened had phage predator loads below the limit of detection (1 plaque forming phage unit (PFU) 15 ml^−1^ concentrated seawater); with maximum plaques per strain of 90 PFU ml^−1^ on day 222; 81 on day 261; 439 on day 286. We note that recovery efficiencies of viruses in iron flocculates resuspended in oxalate solution were not tested for individual samples but have been shown to be approximately 50%^14^ and thus observed PFUs are conservative estimates. Though 28% (125/449) of bacterial isolates on day 222 were *Vibrio tasmaniensis* strains of a single *hsp60* type, only a single isolate with this type was killed by co-occurring phages (10 N.222.48.A2, labeled with a star as a “bloom group”), and no isolates with this *hsp60* type were subsequently isolated. Tree scale of 0.1 substitutions per site indicated by red bars. **b** Strains killed by co-occurring phages (43 plaque-positive on ordinal day 222; 101 on day 261; 141 on day 286) were targeted for genome sequencing and used in subsequent host range assays. Underlying data provided in Supplementary Data 1, see Methods for strain sets analyzed.

Our large-scale bait assay revealed that, at the strain level, lytic phage predation pressure on the majority of coastal ocean *Vibrio* appears low (<134 plaque forming phage L^−1^; limit of detection based on doubled counts assuming 50% recovery efficiency) compared to total virus-like-particle concentrations (10^10^ VLP L^−1^) common in coastal marine environments^15^ (Fig. 1, showing undoubled counts). As individual strains of the most abundant *Vibrio* species in our study typically occur at concentrations of on average <1 cell ml^−1 ^^16^, these findings indicate that encounter rates should be very low between these phages and their hosts. These observations are consistent with previous studies of lytic (plaque forming) phage predator loads on heterotrophic marine bacteria (largely in the family *Vibrionaceae* and genus *Vibrio*^17^, as well as in the genus *Pseudoalteromonas*^18^) by Moebus, which also showed the majority of bacterial strains were subject to 0–10 PFU ml^−1^ in water samples collected in the same year. These observations suggest that mechanisms that increase encounter rates between *Vibrio* phages and their hosts—such as host blooms, spatial structure on small scales, and broad host range - should be important features of phage-bacteria interactions in systems where individual host strains are rare.

### Lytic phage-bacteria interactions within the *Vibrionaceae* are overall sparse and modular

To investigate the host ranges of the phages in this system we purified one phage from each plaque-positive host for further study, representing a final set of 248 independent phage isolates (hosts: Supplementary Fig. 1, phages: Supplementary Data 8). In previous work we showed that these phages represent phylogenetically diverse dsDNA viruses ranging in size from 10 kb – 349kb^19^, including non-tailed members of the recently proposed family *Autolykiviridae*^20^, as well as representatives of the three morphotypes of the Caudovirales (as predicted by Virfam^21^). Host ranges of each of the phages were assayed against a panel of 294 genome-sequenced bacterial strains, including all plaque-positive hosts and 18 additional *Vibrio* strains (selected to represent additional populations of *Vibrionaceae*; for details on these additional strains see Supplementary Data 1 sheet A and filter for all bacterial strains with identifiers without the prefix 10 N). Of these hosts, 279 were lysed by at least one of the 248 phages in the host range assay and were included in subsequent analyses, with genomes of 259 member bacteria and all phages sequenced (Supplementary Data 1).

**Fig. 2:**
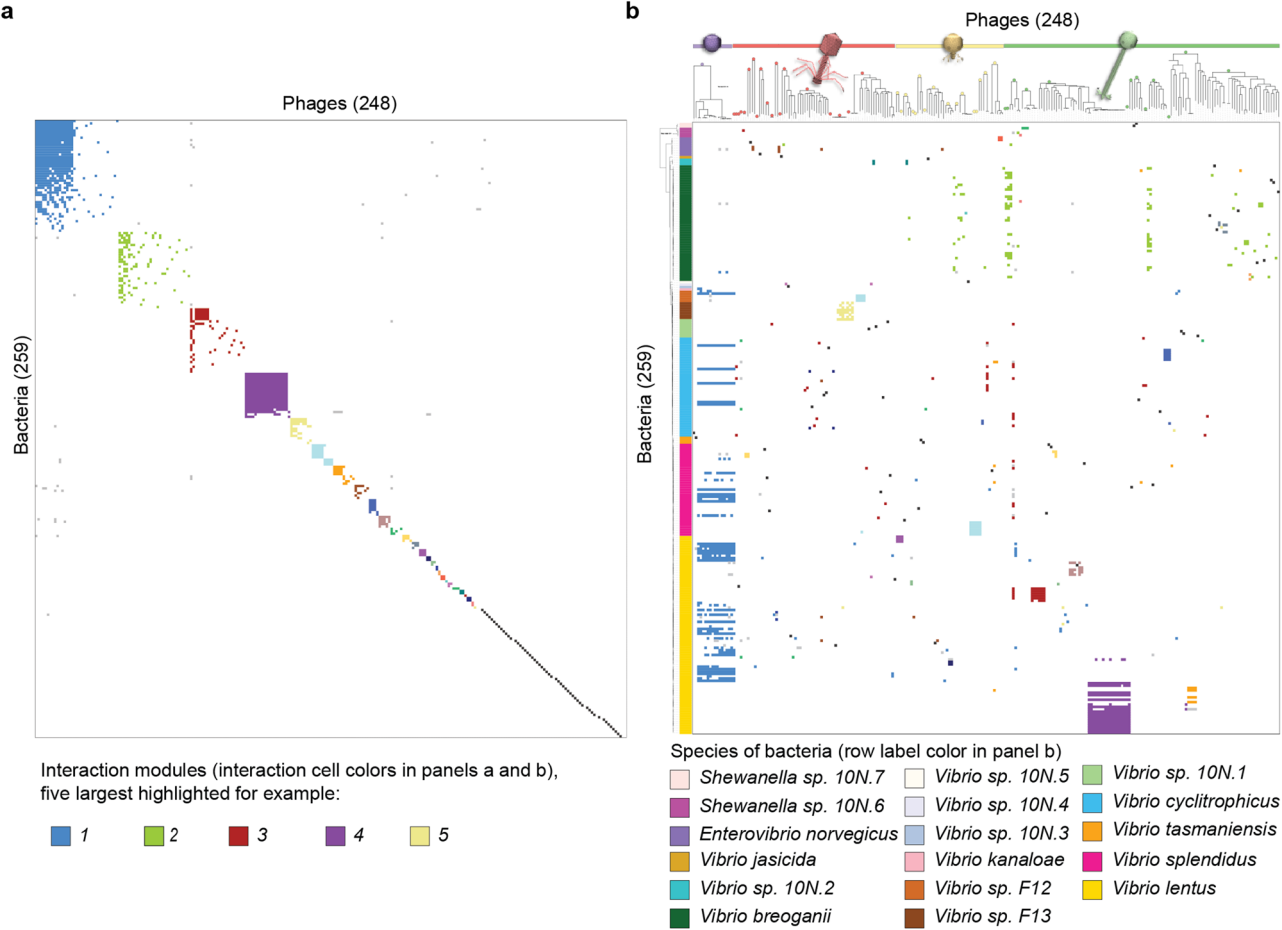
The nested-modular structure of environmental phage–host interaction networks reflects multiple drivers. **a** Network analysis of the Nahant Collection infection matrix shows an overall nested-modular interaction structure and abundance of one-to-one infections. **b** Re-organization of the interactions in light of host phylogenetic and phage genomic diversity reveals that modular structure reflects the influence of host species, phage genera, phage host range strategies, and bloom dynamics. In both panels bacteria are represented as rows and phages as columns; both panels show the same 248 phages and the subset of 259 Nahant Collection hosts which were infected in the host range assay by one of the 248 phages and for which genomes were also available (see details on host subsets in Supplementary Data 1, sheet readme); in both panels a and b, all interactions within each matrix are colored according to BiMat leading eigenvector modules (the five largest groups are shown, for example, as blue, green, red, purple, and light yellow); in panel b bacterial strains are ordered based on phylogeny of concatenated single copy ribosomal protein genes, with leaf colors representing species; in panel b phages are ordered based on manual sorting of VICTOR genus-level trees into groups by morphotype irrespective of their higher order clustering (where VIC-genera of different morphotypes can be intermingled; VICTOR trees represent Genome BLAST Distance Phylogenies (GBDP) based on concatenated protein sequences for each phage genome, with branch lengths representing intergenomic distances scaled in terms of the GBDP distance formula d_6_; each of the 49 phage VIC-genera are represented as a distinct group indicated by a circle filled with the color representing the morphotype of the genus (purple: non-tailed; red: myovirus; yellow: podovirus; green: siphovirus); see Supplementary Data 8 for full original VICTOR phylogeny not sorted by morphotype). Underlying data are provided in Supplementary Data 1 and Source Data Fig. 2, see Methods for strain sets included in the analyses. Phage icon source: ViralZone www.expasy.org/viralzone, Swiss Institute of Bioinformatics. Source Data Fig. 2.

In this large-scale study of the host ranges of 248 phages on 279 hosts, we found that the majority of bacteria were resistant to the majority of phages and that interactions were overall sparse – with only 1436 lytic interactions observed in agar overlay (hereafter “killing”) out of 69,192 possible interactions. We further found that killing interactions were organized in an overall modular fashion – with groups of phages and bacteria clustering into 89 discrete interconnected sets (“modules”, Fig. 2a, subset shown is 248 phages and 259 hosts with sequenced genomes, details in Supplementary Data 1) using the BiMat modularity evaluation methods developed and employed by Flores et al.^22^ to investigate the Moebus-Nattkemper matrix^4,5^. These features of our matrix are strikingly similar to those of the similarly large matrix generated by Moebus and Nattkemper matrix in the 1980s (Table 1). However, unlike this previous matrix, performed at a time when genome sequencing of all members was not possible, we could now also investigate the structure of phage-bacteria interactions in light of genomic and phylogenetic diversity to understand the biological basis of the modular structure observed.

**Table 1:** Comparison of Moebus-Nattkemper and Nahant matrix properties.

Matrix Property	Moebus-Nattkemper Atlantic Time Series Matrix	Nahant Collection Matrix
# of Hosts (H)	286	279
# of Phages (P)	215	248
# of Species (S = H + P)	501	527
# of Interactions (I)	1332	1436
Size (M = H * P)	61,490	69,192
Connectance (C = I/M)	0.02	0.02
Host mean interactions (L_H_ = I/H)	4.7	5.1
Phage mean interactions (L_P_ = I/P)	6.2	5.8
Modularity	0.7950*	0.7306**

Summary of properties using phageSet248 and baxSet279 (see Supplementary Data 1 for further details on isolates included and on infections). *Calculated using the bipartite recursively induced modules algorithm; **calculated using the leading eigenvector algorithm.

### Diverse processes define membership of different interaction modules

The structure of the phage-bacteria interaction network in this study indicates that both broad and narrow host range strategies are important in the coastal marine environment. Whereas the three largest modules represented the majority of lytic interactions observed in agar overlay (53% of all interactions, 768/1436 total infections), the majority of modules were singletons comprised of only a single phage and bacterial strain interacting exclusively with each other (61/89 modules but only 4% of all interactions).

Central to the organization of each of the three largest modules were phages that were able to kill numerous genomically diverse host strains (Fig. 2b, with the three largest modules shown in blue, green, and red fill, respectively; hosts: Supplementary Fig. 1; Supplementary Data 1). The largest module was organized around killing by members of a new family of recently described phages, the *Autolykiviridae*, whose members can infect some but not all host strains in up to 6 species^20^. The second largest module was likewise organized around phages that killed multiple host strains within a single phylogenetically divergent species, *Vibrio breoganii*. This species is non-motile, lives predominantly attached to macroalgal detritus, and is specialized for degradation of algal polysaccharides^23,24^ - and thus is also ecologically distinct from other vibrios. The genomically diverse sipho- and podovirus phages infecting *V. breoganii* hosts were nearly all exclusive to this host species in their infections, suggesting that divergence in bacterial ecology is also reflected in interactions with different groups of phages. The third largest module was organized around a single broad host range siphovirus (1.215.A) that infected 26 host strains in 6 species in our network, including members of both the *Vibrionaceae* and the *Shewanellaceae*. All three of these large modules, while organized around broad host range phages that could infect multiple specific host strains, included other phages that were effectively entrained into the module as a result of sharing a host strain with the module-defining broad host range phages.

The striking dominance of singleton modules in this network highlights the prevalence of exquisitely narrow host range profiles of phages with respect to their local hosts in the coastal marine environment. This finding parallels that of Moebus, who found that for 200 phages isolated from a coastal marine system nearly half infected only the original strain on which they were isolated^25^. Moebus’ work^25^ also suggested that bloom dynamics are likely important in these systems by revealing ephemeral peaks of up to 1500 PFU ml^−1^ that decayed on the order of days. Such high concentrations and dynamic abundances have also been shown in other marine heterotrophic^26^ and cyanobacterial^27,28^ host systems, with observed maxima of up to 36,500 and 35,000 PFU ml^−1^, respectively. The presence in the Nahant matrix of modules that contain multiple closely related phages and hosts isolated on the same days is consistent with a role for host blooms in driving increases in relative abundances of specific phage types. For example, in the 4th largest module (23% of all infections) the majority of phages (18/19) had an average pairwise average nucleotide identity (ANI) of >99%, and infected largely the same set of closely related host strains (18/19 host strains in the module having >99.95% average pairwise ANI). The potential for *Vibrio* to form such blooms is well supported as they proliferate rapidly in response to nutrient pulses and have been observed to rapidly undergo large increases in relative abundance in microbial communities in the environment^11,29^.

### Killing host ranges are not clearly defined by phage morphotypes

As host range breadth has previously been shown to be associated with morphotype^30^, with myoviruses infecting more broadly than other tailed viruses, we examined whether this was true in this dataset. We found that non-tailed viruses infected significantly more strains than tailed viruses, whereas there were smaller differences between tailed viruses. Student’s t-tests showed significant differences in the number of host strains killed by members of the *Autolykiviridae* and each of the three tailed morphotypes—the podoviruses (*p*-value = 4.55e-09), siphoviruses (*p*-value = 1.22e-08), and myoviruses (*p*-value = 3.77e-09); and between myoviruses and siphoviruses (*p*-value = 3.20e-06). By contrast, no significant differences were found between podoviruses and siphoviruses (*p*-value = 0.02), or between podoviruses and myoviruses (*p*-value = 0.04). Autolykiviruses killed on average 31.3 strains (standard deviation 11.3), myoviruses on average 2.0 strains (s.d. 1.6), podoviruses on average 3.2 strains (s.d. 3.7), and siphoviruses on average 5.1 strains (s.d. 6.5), yet there were phages of all morphotypes, including the non-tailed autolykiviruses, that killed host strains in only a single species, in two species, in three or more species, and in two genera (Supplementary Data 2). Three siphoviruses killed hosts in both families of bacteria represented in this study, however, the limited representation of potential non-*Vibrionaceae* hosts precludes any conclusion about whether this reflects a broader pattern. Interestingly, there were no myoviruses that killed the ecologically distinctive *V. breoganii*, though 71/248 phages were of this morphotype and this host species was present on all three isolation days (Fig. 1) and well represented in the host range assay (Supplementary Data 1). Together, these observations indicate that morphotype may not be a reliable indicator of the number of host species a phage will infect but may shape access to hosts with different ecological and habitat associations.

### *Vibrionaceae* phages are diverse and under-sampled

**Fig. 3:**
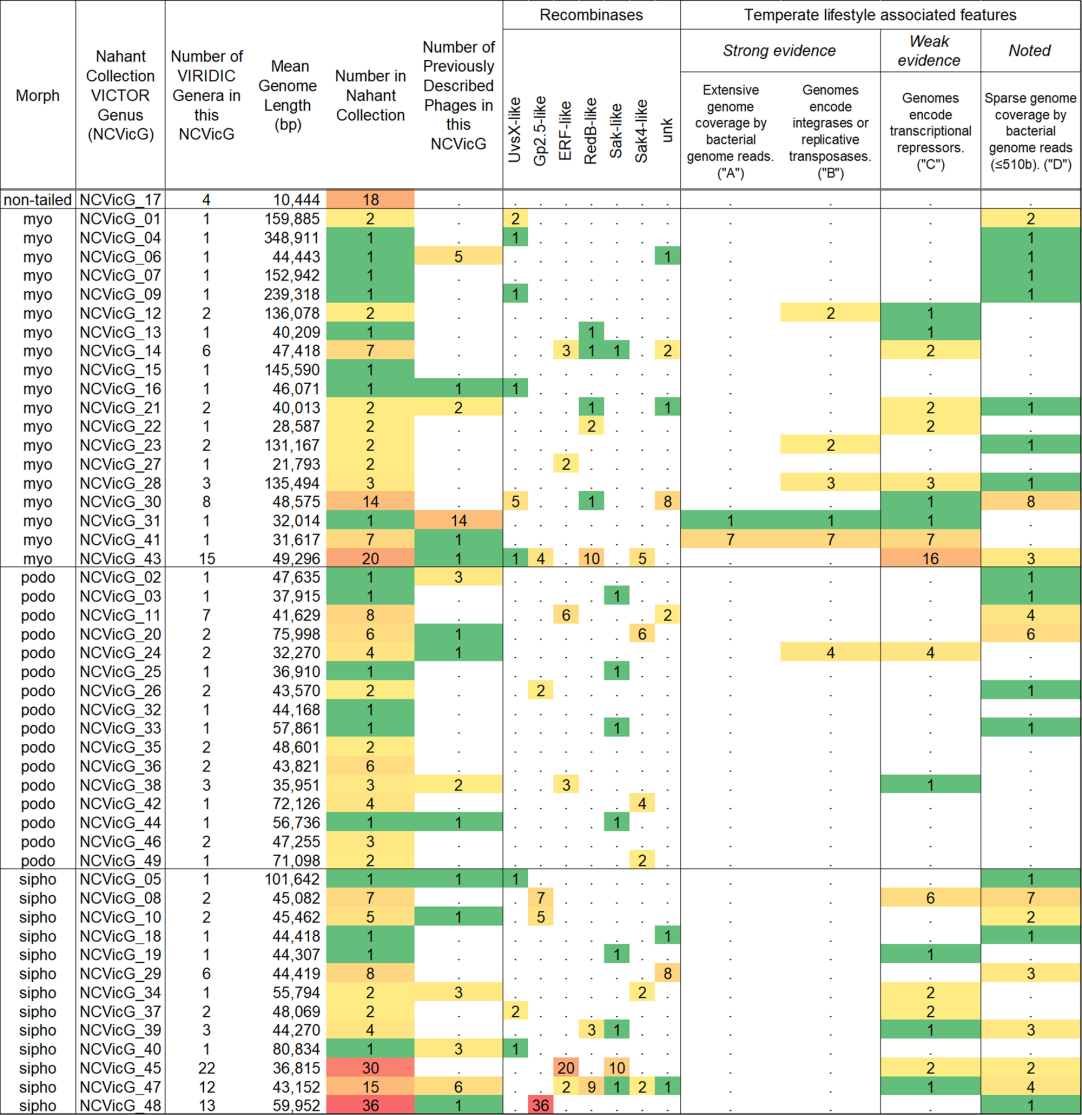
Overview of Nahant Collection phages by VICTOR genus (NCVicG). Features suggestive of temperate life history strategy were evaluated, and findings are highlighted as representing either strong (A and B) or weak (C) evidence, where: A indicates extensive mapping of bacterial genome reads to phage genomes (see Methods); B indicates presence of integrases (PF00239, PF00589) or replicative transposases (PF02914, PF09299); C indicates presence of transcriptional repressors (IPR010982, IPR010744, IPR001387, IPR032499); and D is noted only for reference and indicates sparse mapping of reads from bacterial genomes onto phage genomes (≤510 bases total). Note also that: the phages in NCVicG_17 are representatives of the proposed family *Autolykiviridae*; the sole NCVicG_41 phage is in the described subfamily *Peduovirinae*; the phages in NCVicG_41 encode genes for replicative transposition, and the phages in NCVicG_20, _42, and _49 are N4-like in encoding a giant RNA polymerase. All counts reported in the table for Recombinases and Number in Nahant Collection are based on phageSet248 (see Supplementary Data 1 for strain set lists and descriptors); heat map ranges from 1 (green) to 36 (red).

To next investigate patterns of phage host range across levels of phage genomic diversity, we operationally clustered phages into species and higher order groups. Because a standard approach has not yet been set, we use two methods for identifying groups of more (~species) and less (~genus) closely related groups of phages, VIRIDIC^31^ and VICTOR^32^. VIRIDIC determines intergenomic nucleotide similarities and groups viruses into clusters based on user-defined similarity cut-offs (here defaults of 70% for genera and 95% for species), whereas VICTOR identifies species and genera on the basis of pairwise whole genome distance comparisons followed by clustering benchmarked to previously described viral taxa (here using protein sequences and the d_6_ distance scaling formula). We find that these two methods largely agree at the species level (171 VICTOR species, 188 VIRIDIC species; both VICTOR and VIRIDIC species and genus clusters for each phage indicated in Supplementary Data 1), yet diverge at the genus level (49 VICTOR genera, 151 VIRIDIC genera; VICTOR taxon sequence similarity thresholds highly variable and reported with respect to VIRIDIC intergenomic similarity values in Supplementary Data 3). We provide comparisons between VICTOR and VIRIDIC as supplementary information (Supplementary Fig. 3 and Supplementary Data 3; and see Fig. 3 for overview of VIC-genera and Supplementary Fig. 2 for representation of genera across sampling days). An overview of all phage genomes organized by VICTOR distances are provided in Supplementary Data 8.

To ask whether any of these phage groups include previously described members, we used vConTACT2^33^ to cluster the Nahant Collection phages with >10,722 previously described phages with available genome sequences in NCBI (details in Supplementary Data 4). We found the VICTOR and vConTACT2 genus-level clusters to be largely concordant and identified 17 Nahant Collection VICTOR genera (hereafter VIC-genera or, for species, VIC-species) that include previously described phages, though none in the same VIC-species as Nahant phages. The majority of previously described phages in these VIC-genera also infected hosts in either the *Vibrionaceae* or *Shewanellaceae*, consistent with a previous finding that phage genera are largely specific to host families^32^ (see Supplementary Data 4 for exceptions). We thus find that local phage diversity is overall exceedingly high and under-sampled, even for this well studied host family – with, for example, 51 new VIC-species of phages isolated for one host species (*Vibrio lentus*) across our 3 sampling days.

### Killing host ranges are not defined by phage life history strategy

A small subset of phages in the Nahant Collection show strong evidence for temperate lifestyles (Fig. 3). Phages in 6 of the 49 VIC-genera (VIC-genera 12, 23, 24, 28, 31 and 41; and including all phages in these genera) encode integrases or replicative transposases, and phages in 2 VIC-genera (VIC-genera 31 and 41) were identified as prophages in bacterial genomes. Phages in 20 VIC-genera (including some members of the aforementioned groups) encode transcriptional repressor domains suggestive of potential for temperate life history strategies (see Methods for additional details on annotation of life history strategy and Supplementary Data 5 for read mapping results and summary of phage life history strategy related annotations). A previous study of human microbiome associated coliphages found host ranges of virulent phages to be broader than those of temperate phages^34^, and to likewise evaluate this here we compare host ranges of phages in species within high confidence temperate genera (19/248 phages in 12 VIC-species, Supplementary Data 5) with those in high confidence virulent species (75/248 phages in 35 VIC-species, Supplementary Data 5). Overall, we detect no significant difference in the total number of bacterial species or *hsp60*-types killed by a given phage species in relation to predicted life history strategy (two sample Kolmogorov-Smirnov test for species: D = 0.14524, *p*-value = 0.9917, two-tailed, and for *hsp60*-types: D = 0.2, *p*-value = 0.8671, two-tailed; see Supplementary Data 5 for VIC-species life history assignments).

### Overlap in killing host range is generally common only within phage species, yet recombination occurs more broadly

**Fig. 4:**
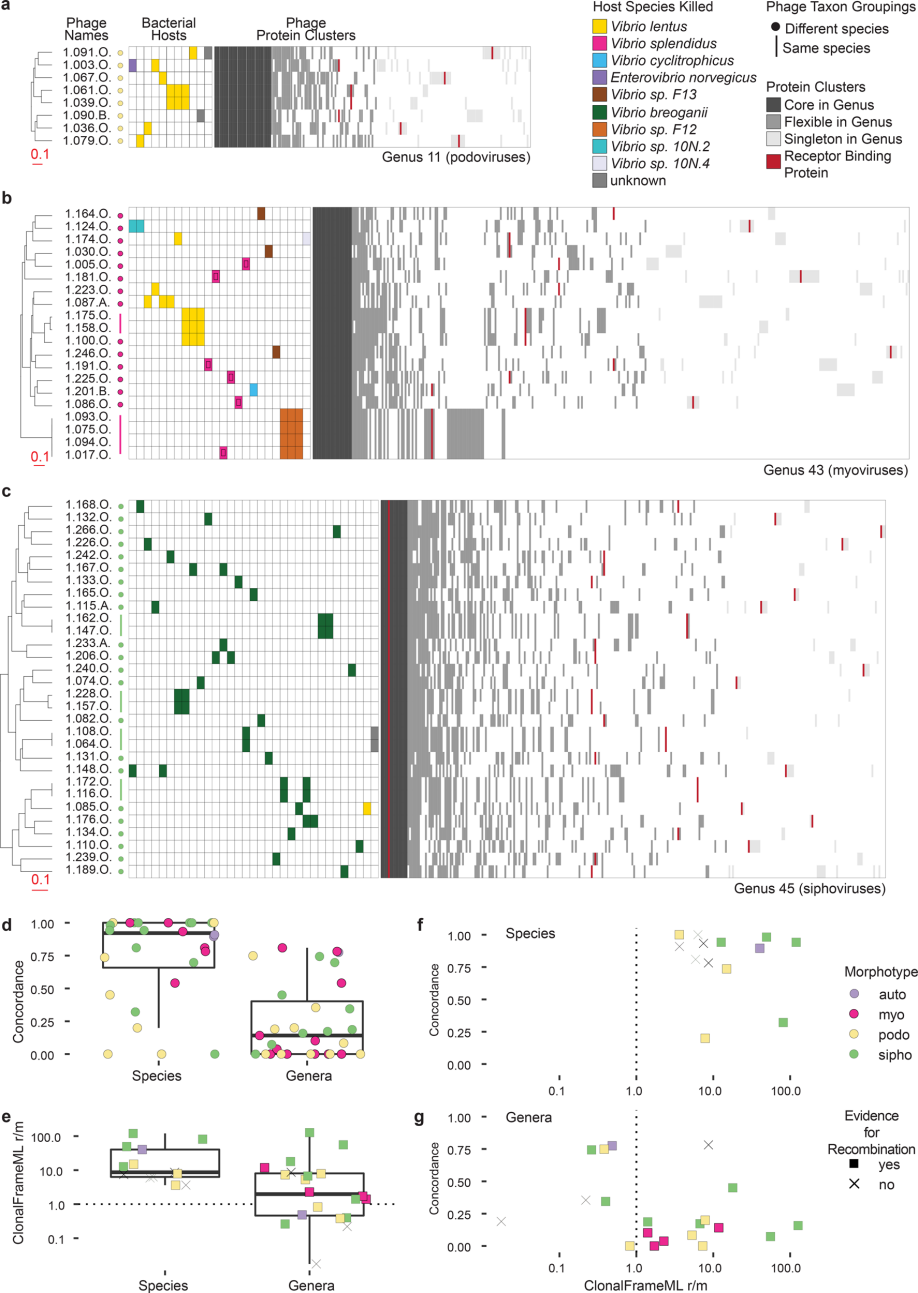
Host range overlap is high within phage VIC-species and low within phage VIC-genera, but recombination occurs both within and between VIC-species. VIC-genus trees for each of a group of podoviruses (**a**), myoviruses (**b**), and siphoviruses (**c**) represent VICTOR Genome BLAST Distance Phylogenies (GBDP) based on concatenated protein sequences for each phage genome, with branch lengths representing intergenomic distances scaled in terms of the GBDP distance formula d_6_ (complete tree with all phages shown in Supplementary Data 8, with underlying data in Newick format provided in Source Data Fig. 2. Filled in cells in the host range matrices aligned to the right of phage names (in rows) show host strains (in columns) killed by each phage. Protein cluster matrices aligned to the right of the host range matrices show all the MMseqs2 protein sequence clusters present in each genus (columns), rank sorted based on the number of phages in the VIC-genus in which they occur. Quantified host range profiles for phages across the collection show that: **d** overlap in killing profiles (concordance) is high within VIC-species (28 VIC-species with ≥2 phages, 105 phages total) but low within VIC-genera (31 VIC-genera with ≥2 phages, including cases of genera represented by only 1 species, 230 phages total; two-sided Welch’s *t*-test *p*-value = 1.45e-07); that **e**, recombination in conserved regions is commonly a greater contributor to genomic diversity in both species and genera (same phage counts as in panel **d**); and, **f** and **g**, that there is no relationship between concordance in killing and recombination for either VIC-species or VIC-genera, respectively. Underlying data and strain information available in Supplementary Data 1 and 6, and in Source Data Fig. 4, see Methods for description of differences in results when considering only single VIC-species representatives in VIC-genus-level analyses. Boxplot features: central line-median; box limits-1st and 3rd quartiles; upper whisker-largest value no larger than 1.5 * IQR (inter-quartile range); lower whisker-smallest value no smaller than 1.5 * IQR. Source Data Fig. 4.

We next considered host range profiles in light of phage species and genera - using VICTOR taxa, as these correspond well with VIRIDIC at the species level but offer greater breadth of diversity at the genus level for systematic supra-species comparisons. We found that whereas overlap in killing is high between phages within VIC-species, it is low between phages in different species. This feature is evident in visual evaluation of host range profiles (infection matrices in Fig. 4a–c), and is consistent with the striking diversity of putative receptor binding proteins (RBPs) among phages of different VIC-species (protein cluster matrices in Fig. 4a–c; Supplementary Data 6). To evaluate these differences quantitatively, we defined a metric of host profile concordance based on Jensen-Shannon distance between host range profiles represented as normalized binary (killed or not-killed) vectors of host strains (see Methods). With this metric, a concordance value of 1 is equivalent to perfect overlap in the host ranges of any two phages, and a value of 0 represents no shared hosts. At the VIC-species level we found that concordance values were generally high (Fig. 4d), with phages in 10 VIC-species showing perfect overlap in their host range, including 3 VIC-species with member phages isolated on different days (this analysis included 105 phages, representing the 28 VIC-species with >1 member; see Source Data Fig. 4 sheet A). By contrast, within VIC-genera, concordance in host range among members was generally low (Fig. 4d), even when calculated using a conservative approach that yields higher estimates of concordance when VIC-species in a VIC-genus contain multiple members (see Methods, see Source Data Fig. 4 for concordances calculated using all members of a genus [sheet B] or with only a single representative of each species [sheet C], and see Supplementary Fig. 4 for scaling of concordance value with size of subsampled groups.).

The differences in host range concordance at the phage VIC-species and VIC-genus levels suggested that there should be corresponding differences in levels of recombination between phage genomes within these groups, yet we found recombination to be occurring both within and between phage VIC-species. We observed this qualitatively in the distributions of flexible genes within VIC-genera, where in some cases nearest-neighbor phages at the whole-genome level share flexible protein cluster genes not with each other, but instead with phages of different VIC-species within their VIC-genus (protein cluster matrices in Fig. 4–c; Supplementary Data 6). To quantitatively evaluate relative contributions of homologous recombination and mutation (r/m) to diversification, we analyzed regions of phage genomes conserved at the VIC-species- and VIC-genus-levels. At the VIC-species level we found that homologous recombination was the greater contributor (r/m > 1; one-sample Wilcoxon test, null hypothesis mean = 1, *p*-value 0.0002) to diversification for the majority of VIC-species with sufficient members to test (Fig. 4e, Source Data Fig. 4 sheet D). This finding of r/m > 1 within phage VIC-species is consistent with similar findings in other environments^35–37^ and suggests that, even in this seemingly rare-encounter opportunity system, phage virion concentrations can reach high enough local concentrations to result in co-infections between members of the same VIC-species in cells of their shared hosts.

Surprisingly we also found a signal for r/m > 1 (one-sample Wilcoxon test, null hypothesis mean = 1, *p*-value 0.0192) at the VIC-genus level (Fig. 4e), even when calculated using a conservative approach that could underestimate this value in VIC-genera containing VIC-species with multiple members (see Methods, Source Data Fig. 4 sheet E). Considering the possibility that this metric could reflect high rates of recombination within only a single species within a genus, we also evaluate r/m within genera using only a single representative of each species within a genus and find that using this approach r/m decreases to <1 for only a single VIC-genus [NCVicG_24] and increases to >1 for a different VIC-genus [NCVicG_17, the *Autolykiviridae*]. It is notable that substituting tools with stricter relatedness thresholds (such as VIRIDIC) to define genera would result in a larger number of quasi-genus groups and that therefore detecting r/m as >1 within VIC-genera is a conservative estimate of the potential extent of homologous recombination occurring between phages in supra-species level taxa.

The importance of recombination at both the VIC-species and VIC-genus levels indicates that overlap in killing between phages is not predictive of their potential for recombination in the context of natural microbial communities. This is corroborated when both quantitative metrics are considered together, which shows a lack of a positive association between host range concordance and r/m on both the VIC-genus (Fig. 4f, Spearman correlation −0.2695786) and VIC-species levels (Fig. 4g, Spearman correlation −0.2417582).

**Fig. 5:**
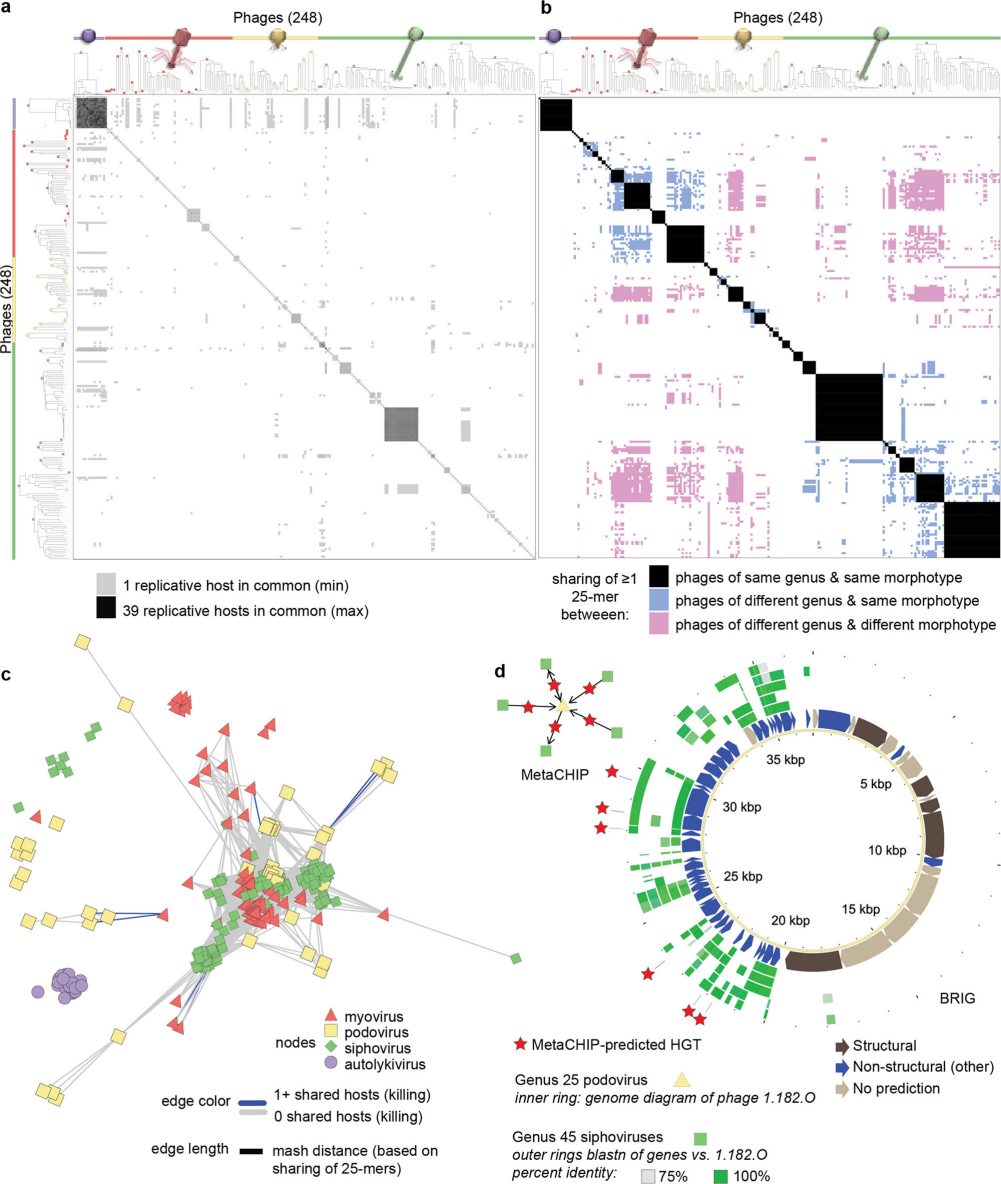
Overlap in killing of host strains is sparse among tailed phages and does not reflect sequence sharing between phages of different VIC-genera or morphotypes. Matrix representations of pairwise comparisons between phages, show that: **a** occurrence of shared replicative hosts, does not reflect **b** occurrence of sharing of ≥1 25-mer between phages of different VIC-genera and morphotypes; in both panels phages are ordered based on manual sorting of VICTOR genus-level trees into groups by morphotype irrespective of their higher order clustering (where VIC-genera of different morphotypes can be intermingled, see Methods for details and Supplementary Data 8 for full original VICTOR phylogeny not sorted by morphotype); each of the 49 VIC-genera are represented as a distinct group indicated by a circle filled with the color representing the morphotype of the VIC-genus (purple: non-tailed; red: myovirus; yellow: podovirus; green: siphovirus). VICTOR trees represent Genome BLAST Distance Phylogenies (GBDP) based on concatenated protein sequences for each phage genome, with branch lengths representing intergenomic distances scaled in terms of the GBDP distance formula d_6_. Extent of sequence sharing that can occur between phage morphotypes and genera that are largely non-overlapping in host killing is revealed by: **c** network visualization of phage genome connectivity by Mash distance, which is defined by sharing of 25-mers; and **d** occurrence of extensive sequence similarity between phages of different morphotypes, as visualized here using Blast Ring Image Generator (BRIG), for which specific directional horizontal gene transfer events can also be detected using MetaCHIP. Source data for panels **a**, **b**, **c**, and **d** are provided in Source Data Fig. 5, see Methods for strain sets included in the analyses; annotations for genes shown in panel **d** are provided in Supplementary Data 6, see Methods for information on methods for defining “structural”, “non-structural” (defined as “other” in Supplementary Data 6), or “no prediction”. Phage icon source: ViralZone www.expasy.org/viralzone, Swiss Institute of Bioinformatics. Source Data Fig. 5.

To understand the potential maximal extent of recent gene flow among all phages in this collection we used a k-mer based approach and found evidence of sequence sharing between phages of different *Caudovirales* morphotypes with non-overlapping host killing. We first used the liberal metric of occurrence of sharing of any 100% identity 25-base pair (25-mer) length sequences between phages, such 25-mers are sufficiently unique in bacterial genomes to be used to recapitulate strain and species level relatedness^38^, and provide a marker for potential recent horizontal gene transfer events^39^. Using this approach, we found far greater potential connectivity of gene flow than suggested by overlaps in host killing (Fig. 5a, b, Source Data Fig. 5). Notably, however, despite extensive overlap in killing host range between autolykiviruses and tailed phages, there were no cases of shared 25-mers between phages in these two groups—a feature consistent with their lack of any shared protein clusters (Supplementary Data 4 and 6).

Considering next the more conservative metric of total numbers of 25-mers shared between any two phage genomes we also found evidence for sequence sharing between divergent tailed phages with non-overlapping host ranges (Fig. 5c, Supplementary Fig. 3); with the maximum number of shared 25-mers between any pair of phages of different morphotypes being 6,169. In the collection, some pairs of phages of different morphotypes show evidence of extensive sequence similarity in non-structural genome regions (e.g. Fig. 5d, similarity between NCVicG45 siphoviruses with the singleton NCVicG25 podovirus), paralleling the observation that the podovirus P22 shares sequence similarity to lambdoid siphoviruses in its non-structural genes^40^; other pairs share only a single 25-mer, for example in a DNA polymerase gene (e.g. in protein cluster mmseq 2145 in podovirus 1.262.O and myovirus 1.063.O, cross-reference Source Data Fig. 5 with shared protein cluster in Supplementary Data 6). Finally, an approach designed to detect specific recent gene transfer events (MetaCHIP^41^) from conserved regions of phage genomes also revealed connections between tailed phages in different VIC-genera, without overlapping host ranges, and between which there were often long regions of high genomic sequence similarity (Fig. 5d, Supplementary Fig. 5). Overall, however, when considering only VICTOR distances between phages (based on whole genome concatenated protein sequences), short pairwise distances occur almost exclusively between phages of the same morphotype whereas large pairwise distances can be observed both within and between phage morphotypes (Supplementary Fig. 6).

Altogether, these observations on the extent of recombination among tailed phages despite their lack of overlap in killing suggests that these phages are generally infecting more hosts than they are able to kill. This is supported by a recent study of this collection showing that phages in different VIC-genera can infect the same sets of closely related host strains using different receptors. While these receptors are highly monomorphic at the bacterial species/population level, killing profiles are non-overlapping due to differential bacterial carriage of highly specific phage defense systems (Hussain and Dubert et al.^42^, where the “orange” phages in the reference are VIC-species 165 and 99 in VIC-genus 47, and the “purple” phages are VIC-species 144 in VIC-genus 48). Thus, co-infections of host cells by multiple different phages (necessary to allow for recombination between phage genomes) are likely far more common in natural communities than predicted by killing host ranges.

### Nucleotide sharing between phages can lead to cross-mapping of sequencing reads

Considering the potential for widespread sequence sharing to influence mapping of viral reads to reference genomes, we investigated cross-mapping using a recently developed rapid k-mer based pseudo-alignment approach^43^. We found that cross-mapping of reads can occur between phages of different VIC-species, VIC-genera, and morphotypes within this collection. False positive classifications of reference presence were reduced when using shorter simulated read lengths as a result of overall lower collateral (false positive) sequence coverage when the basis for the mapping was a single 31-mer match in the sequence (Supplementary Fig. 7, see Methods). This observed potential for cross-mapping calls for a cautious approach in using read-mapping to reference genomes in determining whether specific phages are present in metagenomic samples or predicting which hosts virus pools are interacting with.

### Recombinases are prevalent in *Vibrionaceae* phage genomes

**Fig. 6:**
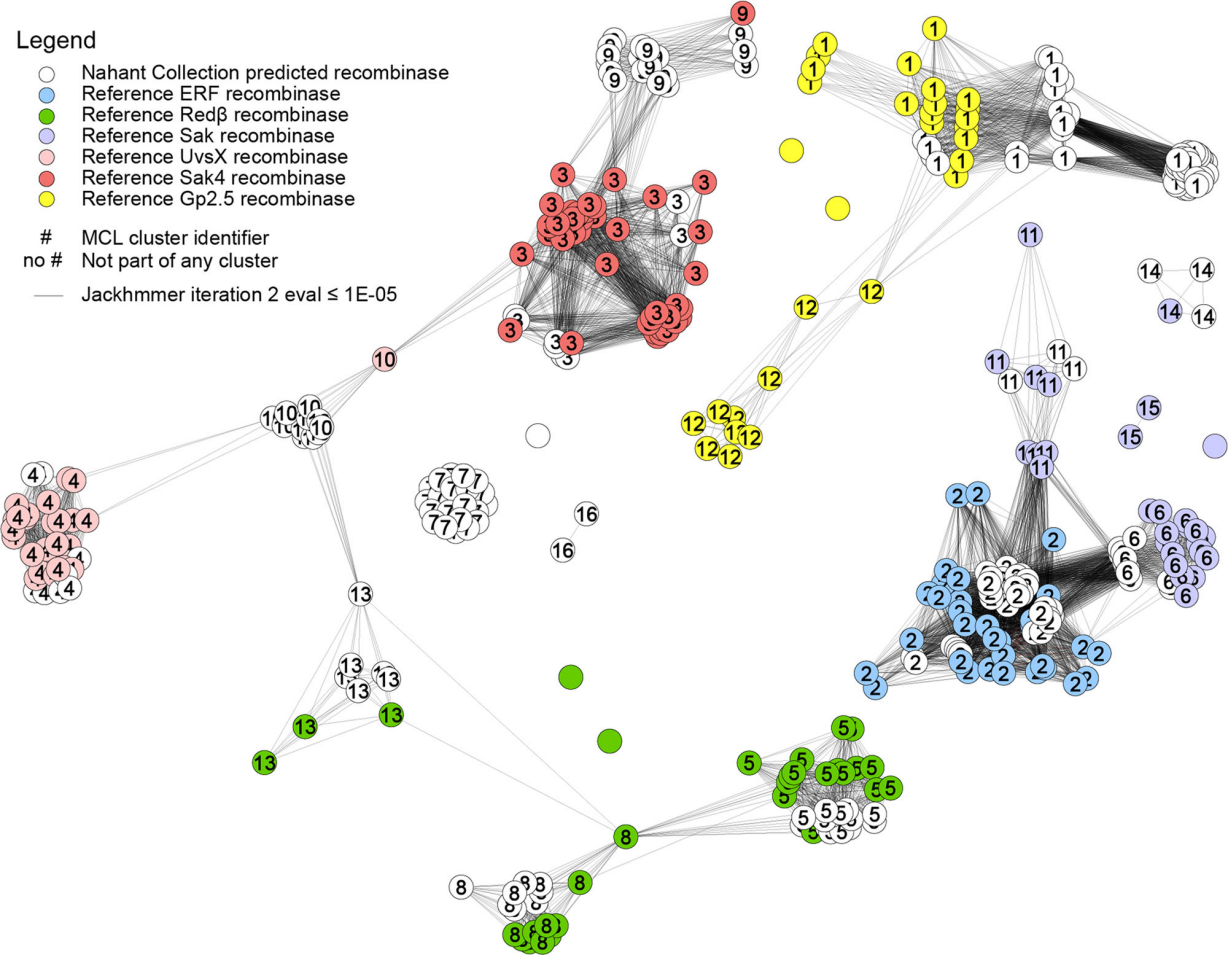
Homologs of Rad52-, Rad51-, and Gp2.5-superfamily recombinases are present in Nahant Collection phages. Iterative HMM searches against Nahant phage protein sequences using 194 reference seed sequences representing 6 families of recombinases (ERF-, Redβ-, Sak-, UvsX-, Sak4-, Gp2.5-like) identified 156 homologs in the collection. Annotation of these 156 genes in the Nahant phage genomes allowed discovery of an additional 4 protein sequence clusters as also being putative recombinases on the basis of shared genome position in related phages, and presence of nearby recombinase-associated exonucleases. Putative Nahant recombinases were assigned to families following MCL-based clustering on the basis of co-clustering with previously described representatives, this allowed for assignment of all but 24 of the 224 Nahant phage sequences. Two clusters of Nahant recombinases (MCL cluster 7 and MCL cluster 16), as well as one unclustered singleton sequence, could not be linked to known families in the network and thus may be representatives of new families of recombinases. In the figure, nodes represent phage protein sequences and are colored by whether they are Nahant phage sequences (white) or reference sequences drawn from Lopes et al. (colors); edges represent sequence pairs with an e-value of < 1e-05 in the second iteration of a jackhmmer search; figure generated using Cytoscape with the Edge-weighted Spring Embedded Layout based on the jackhmmer score; node labels represent MCL cluster as determined using the ClusterMaker2v.1.2.1 Cytoscape plugin with the MCL cluster option and all settings at default and granularity set to 2.5. Underlying data are provided in Supplementary Data 7 and predicted recombinase genes are highlighted in genome diagrams in Supplementary Data 9).

The overall prevalence of sequence sharing observed between phage genomes suggests that they harbor homologous recombination systems, and indeed we find that recombinase genes are encoded by the majority of tailed phages in this collection. Low fidelity single strand annealing protein (SSAP) based recombinase systems such as those in the Rad52-superfamily (e.g. Redβ/RecT-, ERF-, and Sak-families) are common in temperate phages^44^, and are thought to play an important role in their extensive genome modularity and mosaicism^45^. Such recombinases have been shown to be associated with large-scale recombination events of up to 79% genome length between incoming temperate phages and resident prophages^46^ and are useful tools for in vitro genetic engineering (recombineering)^47–50^ as they can facilitate recombination between sequence regions with as little as 23-bp sequence identity^51^. Noting a number of putative SSAP recombinase genes in our initial annotations, we sought to more systematically evaluate their representation in our diverse collection of phages. Using representative sequences^44^ as seeds for iterative searches, followed by gene neighborhood analysis, we identified putative recombinases in 196/230 (85%) tailed phages (in phageSet248, see Methods and Supplementary Data 8 showing genome diagrams with recombinases highlighted), with 117 of these resembling low-fidelity Rad52-superfamily and Sak4-like Rad51-superfamily recombinases commonly associated almost exclusively with temperate phages^44^ (Fig. 6, Supplementary Data 7). That no recombinases were identified among the autolykiviruses, though these viruses also showed evidence for high rates of intra-species recombination (when calculated using single representatives of each species rather than all members, Source Data Fig. 4, sheet F), indicates distinct pathways underlying observed recombination in this group. These results suggest that just as horizontal gene transfer in microbial communities may allow bacteria to evolve resistance to phages, recombination and genetic exchange between phages may likewise be important in overcoming this resistance.

## Discussion

The findings of this work appear at first contradictory: that recombined phage genomes commonly co-exist in natural microbial communities, yet overlap in phage killing in the context of natural diversity is rare. Previous work has spoken to the importance of recombination in generation of phage diversity globally and locally^52^, and our observations imply that recombinants are generated in co-infections of shared hosts that are more frequent than are revealed by killing assays. That these largely unobserved implied co-infections are occurring in recent evolutionary and ecological time is indicated by the higher contribution of homologous recombination than mutation to sequence divergence in many phage groups in our dataset.

We propose two main complementary model scenarios to unify and reconcile these apparently contradictory observations – one addressing trade-offs between growth and phage defense in bacteria and the other pointing to recombination as an important mechanism for phage survival in the face of selective pressure by bacterial anti-phage defenses.

**Fig. 7:**
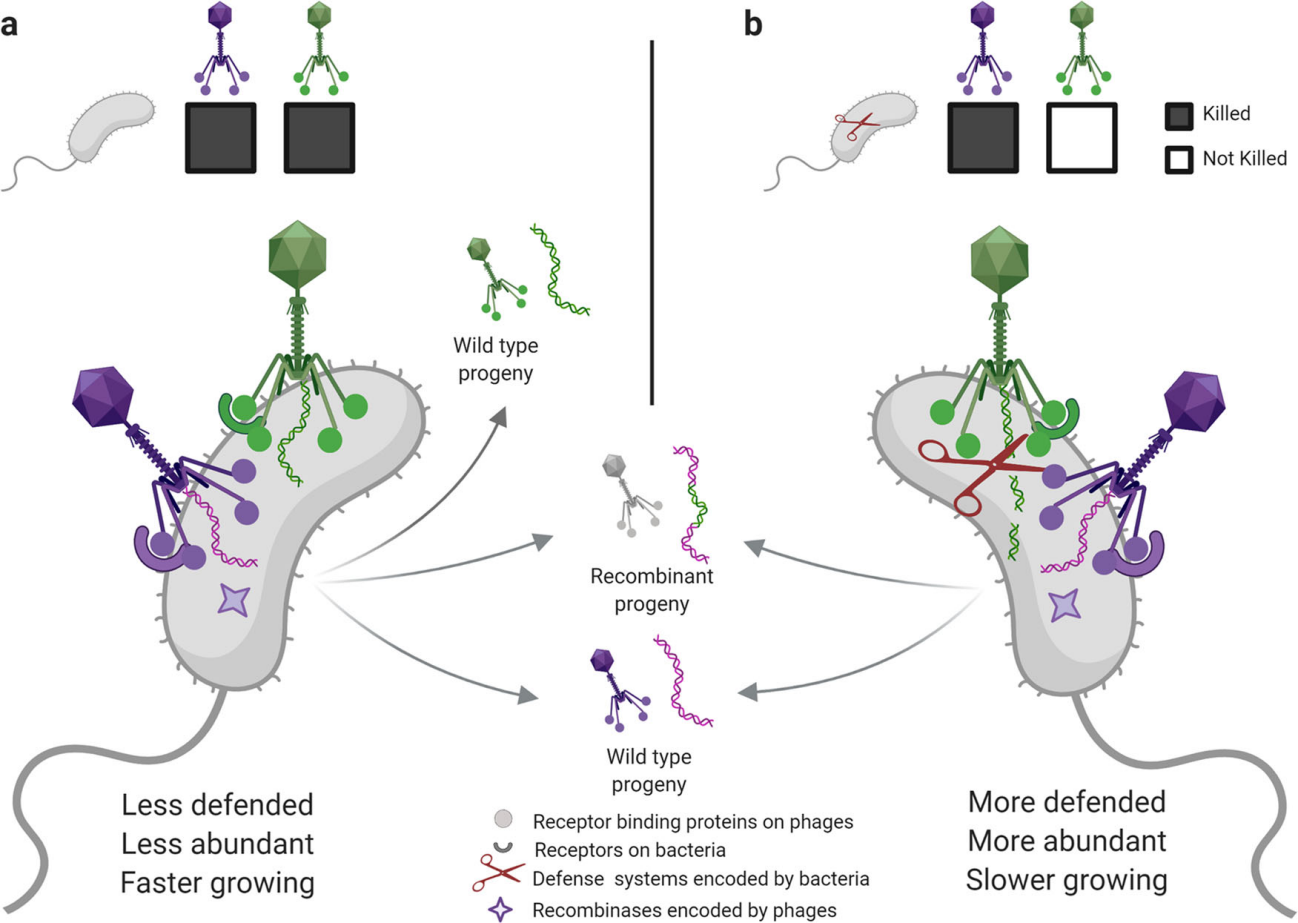
Cryptic-coinfection models reconcile sparse killing overlap with prevalent recombination in phages. We propose that two classes of cryptic (as in rarely observed in the laboratory) co-infections are key in unifying two at first contradictory observations - that in natural microbial communities overlap in killing is rare among tailed phages, yet recombinant phage genomes are common. First **a**, co-infections in rarely observed broadly sensitive hosts killed by multiple phage types (indicated by filled in squares in the figure) may be an important source of phage recombinants in local microbial communities. Where there is an inverse relationship between phage sensitivity and relative abundance and growth rate, broadly sensitive host strains may be ecologically important but systematically underrepresented in isolation studies. Second **b**, co-infections in commonly observed defended hosts that are only killed by a few phage types (indicated by both filled in and empty squares) may also be an important source of recombinant phage progeny, particularly where phage encoded recombinases are expressed in the presence of phage genome fragments generated by host anti-phage defense systems. In both scenarios, where phages are associated with hosts through states of lysogeny or pseudolysogeny, the probability of co-infections, and thus potential for generation of recombinants, will be even greater. Figure created with BioRender.com.

First (Fig. 7a), recombinant phages may be disproportionately generated during co-infections in broadly sensitive (killed) hosts. Previous work with the marine phototroph *Synechoccocus* by Waterbury and Valois^27^ showed that the rarest bacterial strains were the fastest growing as well as the most sensitive to phages. As a result of their lower relative abundances in the environment, these ecologically important broadly phage sensitive hosts may thus be underrepresented in cultivated isolate collections such as ours. Recent work^42^ has also demonstrated that rapid turnover in anti-phage defense systems^42^ is key to fine-scale differences in phage sensitivity among closely related bacterial strains, and to what extent this rapid turnover transiently yields rare broadly sensitive hosts susceptible to co-infection and killing by multiple phage types remains to be determined.

Second (Fig. 7b), penetrative host ranges of phages^53^ are likely generally substantially broader than the replicative host ranges revealed by killing assays like those used in our study – with narrow host ranges reflecting effective anti-phage defense systems rather than lack of phage adsorption and genome delivery. This has recently been shown to be true for phages in this collection in members of VIC-genera 47 and 48, the “orange” and “purple” phages in Hussain and Dubert et al.^42^. Previous studies in marine *Synechococcus* and *Prochlorococcus*^54^, in *E. coli*^55^, in *Mycobacterium*^56^, and in diverse other groups of bacteria, have also shown that phage penetrative host ranges are often broader than replicative host ranges^54^. Findings that highly sensitive indicator strains can yield very high local phage predator loads in both heterotrophic and cyanobacterial host systems further support that local specificity of interactions reflects local defenses against phages, rather than general lack of adsorptive hosts. Altogether, this model is supported by work showing that anti-phage defense systems that effectively abrogate replication^57^ are widespread, diverse, and patchily distributed among strains within bacterial species^58^. Indeed, having broad infective host ranges that expose phages to nucleic acid degrading host defense systems may select for carriage of recombinase genes that facilitate rescue in what would otherwise be abrogated infections.

In both model scenarios, the potential for co-infections and recombination are expected to be shaped also by both phage and bacterial ecology. Conditions and phage life history strategies enriching for lysogeny or pseudolysogeny will increase potential for recombination events^59^, and infections at high cell densities, such as on particles and in biofilms may likewise increase co-infections and thus potential for recombination. The *Vibrio* populations in this study included both predominantly attached and generalist groups with free-living members, and the majority of their phages lack integrases and are not detected as prophages - to what degree cryptic co-infections contribute to recombination in phages of predominantly free-living groups with few associated prophages, such as *Prochlorococcus* and *Pelagibacter*, remains to be explored.

Other scenarios and mechanisms may also be important for giving rise to patterns of recombination in the absence of predicted host overlap like those we observe. For example, where phages are able to replicate yet do not readily form plaques^60^ under selected laboratory conditions, the potential for co-infections will also be higher than predicted by plaque assay based study. And, where bacterial genomes harbor resident prophages, these may offer intermediate reservoirs facilitating gene flow between exogenous phages that do not overlap temporally in their infections of the host.

Overall, the importance of cryptic co-infections indicated by our results underscores the importance of considering that assays of killing host ranges are not predictive of the potential for diffusion of exogenous phage genes into local phage genome pools when they are introduced for bioengineering and therapeutic applications.

## Methods

### Sampling

#### Environmental sampling

Samples were collected from the littoral marine zone at Canoe Cove, Nahant, Massachusetts, USA, on 22 August (ordinal day 222), 18 September (261) and 13 October (286) 2010, during the course of the three month Nahant Collection Time Series sampling^11^.

### Bacterial isolation and characterization

#### Bacterial isolation

Bacterial strains were isolated from water samples using a fractionation-based approach^7^ as previously described^19,20^. In brief, seawater was passed first through a 63um plankton net and then sequentially through 5um (Whatman 111113 or Sterlitech PCT5047100), 1um (Whatman 111110 or Sterlitech PCT1047100), and 0.2um (Whatman 111106) hydrophilic polycarbonate filters; material recovered on the filters was resuspended by shaking for 20 min; dilution series of resuspended cells were filtered onto 0.2um polyethersulfone filters (Pall 66234) in a carrier solution of artificial seawater (40 g Sigma Sea Salts, S9883; 0.2um filtered), and filters placed directly onto *Vibrio*-selective MTCBS plates (BD Difco TCBS Agar 265020, supplemented with with 10 g NaCl per liter to 2% final w/v). Colonies (96) from each of three replicates of each size fraction were selected from the dilution plates with the fewest numbers of colonies (1,152 isolates per isolation day). Colonies were purified by serial passage, first onto TSB-II (Difco Tryptic Soy Broth, 1.5% BD Difco Bacto Agar 214010, amended with 15 g NaCl to 2% w/v), second onto MTCBS, finally onto TSB-II again. Colonies were inoculated into 1 ml of 2216 Marine Broth (BD Difco 279110) in 96-well 2 ml culture blocks and allowed to grow, shaking at room temperature, for 48 h. Glycerol stocks were prepared by combining 100 ul of culture with 100 ul of 50% glycerol (BDH 1172-4LP) in 96-well microtiter plates and sealed with adhesive aluminum foil for preservation at −80 °C.

#### Bacterial *hsp60* gene sequencing

To obtain *hsp60* gene sequences for isolates, Lyse and Go (LNG) (Pierce, Thermo Scientific 78882) treatments of subsamples of the same overnights cultures used in the bait assay (described below) were used directly as template in PCR amplification reactions. PCR reactions were prepared in 30 ul volumes, as follows: 1 ul LNG template, 3 ul 10x buffer, 3 ul 2 mM dNTPs, 3 ul 2um *hsp60*-F primer, 3 ul 2um *hsp60*-R primer, 0.3 ul NEB Taq, 16.7 ul PCR-grade HOH; with *hsp60*-F (H279) primer sequence: 5′-GAA TTC GAI III GCI GGI GAY GGI ACI ACI AC-3′, and* hsp60*-R (H280) primer sequence: 5′-CGC GGG ATC CYK IYK ITC ICC RAA ICC IGG IGC YTT-3′^61^ (Supplementary Table 1). PCR thermocycling conditions were as follows: initial denaturation at 94 °C for 2 min; 35 cycles of 94 °C for 1 min, 37 C for 1 min, 72 °C for 1 min; final annealing at 72 °C for 6 min; hold at 10 °C. PCR products were cleaned up by isopropyl alcohol (IPA) precipitation, as follows: addition of 100 ul 75% IPA to 30 ul PCR reaction product, gentle inversion mixing followed by 25 min incubation at RT, 30 min centrifugation at 2800 rcf, addition of 50 ul 70% IPA with gentle inversion wash, centrifugation at 2000 rcf, inversion on paper towels to remove IPA, 10 min centrifugation at 700 rcf, air drying in PCR hood for 30 min, resuspension in 30 ul PCR HOH. PCR products were Sanger sequenced (Genewiz, Inc.) using *hsp60*-R primer, as follows: 5 ul of 5 um *hsp60-*R primer, 7 ul nuclease free water, 3 ul DNA template. For a subset of strains *hsp60* sequences were obtained from subsequently determined whole-genome sequences. *Hsp60* sequences were aligned to the *hsp60* sequence previously published for *Vibrio* 1S_84 and trimmed to 422 bases using Geneious (https://www.geneious.com/). Accession numbers for these 1287 strains are provided in Supplementary Data 1, where they are identified as baxSet1287.

#### Bacterial *hsp60* phylogenies

A phylogenetic tree of relationships among bacterial isolates screened in the bait assay (described below) was produced based on a 422 bp fragment of the *hsp60* gene, derived either from Sanger or whole genome sequences; with *E. coli* K12 serving as the outgroup. Sequences from each of the three days of isolation were aligned using muscle v.3.8.31^62^ with default settings (*muscle -in $seqsALL -out $seqsALL.muscleAln*), and a single tree including all 1287 sequences from all the days was generated using FastTree v.2.1.8^63^ (*FastTree -gtr -gamma -nt -spr 4 -slow* < *$seqsALL.muscleAln* > *$seqsALL.muscleAln.fasttree*). For presentation in Fig. 1 three sub-trees including only nodes from each day were produced using PareTree v.1.0.2^64^ (*java -jar PareTree1.0.2.jar -t O -del notDay222.txt -f $seqsALL.$round.muscleAln.fasttree.DAY222*). Trees were visualized using iTOL^65^ and painted with metadata for each of the strains, including: sensitivity to killing in agar overlay by co-occurring phage predators collected on the same day and, for the subset of strains that were genome sequenced and also included in the host range matrix, the bacterial species, based on concatenated ribosomal protein analysis using RiboTree^66^ as described below. Isolation days for each of the strains included in these analyses are provided in Supplementary Data 1, where these strains are identified as baxSet1287.

#### Bacterial genome sequencing and assignment to populations

To assign genome-sequenced bacterial isolates used in the host range assay to species, we use the RiboTree tool^66^ to produce a phylogeny based on concatenated single copy ribosomal proteins as in^23^. We include strains of previously described *Vibrionaceae* in preliminary analyses as reference strains and assign species names to new isolates based on clustering with named representatives, as well as provide placeholder names for newly identified clades with no previously described representatives. Trees were visualized using iTOL^65^ and the representation including only those strains included in the host range assay is shown in Supplementary Fig. 1; population assignments and accession numbers for this set of 294 genomes, which also includes a small number of previously isolated bacterial strains that were included in the host range assay (described below), are provided in Supplementary Data 1, where they are identified as baxSet294.

### Viral isolation and characterization

We have previously described features of the viruses of the Nahant Collection^20^, as well as approaches used for the standardization of their genome assemblies^19^, additional details are provided below.

#### Viral sample collection

The iron chloride flocculation approach was used to generate 1000-fold concentrated viral samples from 0.2 um-filtered seawater, as follows. For each isolation day, triplicate 4 L seawater samples were filtered through 0.2 um polyethersulfone cartridge filters (Millipore, Sterivex, SVGP0150) into collection bottles, spiked with 400 uL of FeCl_3_ solution (10 gL^−1^ Fe; as 4.83 g FeCl_3_•6H_2_O (Mallinckrodt 5029) into 100 ml H_2_O), and allowed to incubate at room temperature for at least 1 h. Virus-containing flocs were then recovered from the sample by filtration onto 90 mm 0.2 um polycarbonate filters (Millipore, Isopore, GTTP09030) under gentle vacuum in a 90 mm glass cup-frit system (e.g Kontes funnel 953755-0090, fritted base 953752-0090, and clamp 953753-0090); once liquid was fully passed, the funnel was removed and, with the vacuum pump left on, the filters were folded into quarters, removed from the fritted base, and inserted into a 7 ml borosilicate glass vial. A volume of 4 ml of oxalate-EDTA solution (prepared from stock solution as 10 ml 2 M Mg_2_EDTA (J.T. Baker, JTL701-5), 10 ml 2.5 M Tris-HCl (Promega PAH5123), 25 ml 1 M oxalic acid (Mallinckrodt 2752); adjusted to pH 6 with 10 M NaOH (J.T. Baker, 3722-01); final volume 100 ml; used within 7 days of preparation and maintained at room temperature in the dark) was added to the vial and the sample allowed to dissolve at room temperature for at least 30 min before transfer to storage at 4 °C. A reagent used in this original formulation (JT-Baker 7501 Mg_2_EDTA) is no longer available and an updated recipe is provided elsewhere^67^.

#### Bait assay and associated viral plaque archival

In order to obtain estimates of co-occurring phage predator loads at bacterial strain level resolution, and generate plaques from which to isolate phages, we exposed 1440 purified bacterial isolates to phage concentrates from their same day of isolation (1334 yielded lawns sufficient to evaluate for plaques, and *hsp60* sequences could be determined for 1287 of these). Bacterial strains screened included 480 isolates from each ordinal day, representing 120 strains from each of 4 size-fractionation classes (0.2 um, 1.0 um, 5.0 um, 63 um) details of isolation origin are provided for each strain in Supplementary Data 1, and description of naming conventions is as previously described^19^. For the bait assay each strain was mixed in agar overlay with seawater concentrates containing viruses (15 ul concentrate, equivalent to 15 ml unconcentrated seawater assuming 100% recovery efficiency; derived from pooling of three replicate virus concentrates from each day). We note that recoveries were not tested for individual samples and that previous tests^14^ of recovery efficiency have shown that resuspension of iron flocculates in oxalate solution yields initial recoveries of approximately 50% (49 ± 3% and 55 ± 11% for a marine sipho- and myo-virus respectively, at 24 h post re-suspension) and shows low decay rate over time (47 ± 5% and 73 ± 16% for a marine sipho- and myo-virus respectively, at 38 days post re-suspension). All of our assays were performed approximately 8–9 months post-sampling from oxalate concentrates stored at 4 °C. Agar overlays were performed based on the previously described Tube-free method^13^, as follows. Bacterial strains were prepared for agar overlay plating by streaking out from glycerol stocks onto 2216MB agar plates with 1.5% agar (Difco, BD Bacto, 214010), and allowed to grow for 2 days at room temperature. Strains were then inoculated into 1 ml 2216MB in a 96-well culture block and incubated 24 h at room temperature shaking at 275 rpm on a VWR DS500E orbital shaker. Immediately prior to use in direct plating the OD600 was measured in 96-well microtiter plates and subsamples were taken for Lyse and Go (LNG) processing for DNA (10 ul culture, 10 ul LNG). Phage concentrates were prepared for plating by pooling 1.2 ml from each of the concentrate replicates into a 7 ml borosilicate scintillation vial. Cultures were transferred from overnight culture blocks to 96-well PCR plates in 100 ul volume and 15 ul of pooled phage concentrate was added to cultures one row at a time, with each row plated in agar overlay before adding phage concentrate to the next row of bacterial cultures. Mixed samples of 100 ul bacterial overnight culture and 15 ul pooled phage concentrate were transferred to the surface of bottom agar plates (2216MB, 1% agar, 5% glycerol, 125 ml L^−1^ of chitin supplement [40 g L^−1^ coarsely ground chitin, autoclaved, 0.2 um filtered]). A 2.5 ml volume of 52 °C molten top agar (2216MB, 0.4% agar, 5% glycerol BDH 1172-4LP) was added to the surface of the bottom agar and swirled around to incorporate and evenly disperse the mixed bacterial and phage sample into an agar overlay lawn. Agar overlay lawns were held at room temperature for 14–16 days and observed for plaque formation. Glycerol was incorporated into this assay to facilitate detection of plaques^68^. Chitin supplement was incorporated into this assay to facilitate detection of phages interacting with receptors upregulated in response to chitin degradation products. A variety of preliminary tests exploring potential optimizations to agar compositions for direct plating indicated that the addition of chitin did not negatively impact recovery of plaques with control phage strains tested. After approximately 2 weeks, plaques on agar overlay lawns were cataloged and described with respect to plaque morphology and plaques were picked for storage based on the previously described Archiving Plaques method^13^, as follows. All plaques were archived from plates containing less than 25 plaques, on plates with larger numbers of plaques a random subsample of plaques from each distinct morphology were archived. A polypropylene 96-well PCR plate was filled with 200 ul aliquots of 0.2 um filtered 2216MB, agar plugs were collected from plates using a 1 ml barrier pipette tip and ejected into the 2216MB, skipping one well between each sample to minimize potential for cross-contamination, for a final count of 48 phage plugs per plate. Plaque plugs were soaked at 4 °C for several hours to allow elution of phage particles into the media. After soaking, 96-well plates were centrifuged at 2,000 rcf for 3 min before proceeding to the next step. Plug soaks were then processed for two independent storage treatments. For storage at 4 °C, plates were processed by transferring 150 ul of eluate from each well to a 0.2 um filtration plate (Millipore, Multiscreen HTS GV 0.22um Filter Plate MSGVS22) and gently filtered under vacuum to remove bacteria, the cell-free filtrates containing eluted phage particles from each plaque plug were stored at 4 °C. For storage at −20 °C, 50 ul of 50% glycerol was added to the residual ~50 ul of the plug elution, often still containing the agar plug. In this way all plaques were characterized and many plaques from each strain were archived in two independent sets of conditions. Total plaque counts for all strains included in the bait assay are represented in Fig. 1, and provided in Supplementary Data 1, where they are identified as baxSet1287. Notes on limitations to the assay: Water temperatures on each of the three isolations days were 13.8 °C, 16.3 °C, and 14.2 °C, for days 222, 261, and 286; as bait assays were performed at room temperature (approximately 22 °C) some phages requiring lower temperatures may not have yielded plaques. The majority of plates were evaluated for plaque formation twice, on day 1 and day 13, thus any plaques appearing after day 1 and disappearing before day 13 – for example as a result of overgrowth of lysogens—are likely to have been missed in these assays.

#### Viral purification

A subset of plaques archived during the bait assay was selected for phage purification, genome sequencing, and host range characterization. This subset included single randomly-selected representatives from each plaque-positive bacterial strain. Minor details of the purification and lysate preparation varied across samples but were largely as follows. Phages were purified from inocula derived primarily from −20 °C plaque archives, and secondarily from 4 °C archives when primary attempts with −20 °C stocks failed to produce plaques. Three serial passages were performed using Molten Streaking for Singles^13^ method. Agar overlay lawns for passages were prepared by aliquoting 100 ul of host overnight culture (4 ml 2216MB, colony inoculum from streak on 2216MB with 1.5% Bacto Agar, shaken overnight at RT at 250 rpm on VWR DS500E orbital shaker) onto a standard size bottom agar plate and adding 2.5 ml of molten 52 °C top agar as in the bait assay, swirling to disperse the host into the top agar and form a lawn, and streaking-in phage with a toothpick either from the plaque archive or directly from well-separated plaques in overlays from the previous step in serial purification. Following plaque formation on the third serial passage plate plaque plugs were picked using barrier tip 1 ml pipettes and ejected into 250 ul of 2216MB to elute overnight at 4 °C. Plaque eluates were spiked with 20 ul of host culture and grown with shaking for several hours to generate a primary small-scale lysate. Small scale primary lysates were centrifuged to pellet cells and titered by drop spot assay to estimate optimal inoculum volume to achieve confluent lysis in a 150 mm agar overlay plate lysate. Plate lysates were generated by mixing 250 ul of overnight host culture with primary lysate and plating in 7.5 ml agar overlay. After development of confluent lysis of lawns as compared against negative control without phage addition, the lysates were harvested by addition of 25 ml of 2216MB, shredding of the agar overlay with a dowel, and collection of the broth and top agar. Freshly harvested lysates were stored at 4 °C overnight for elution of phage particles, the following day lysates were centrifuged at 5,000 rcf for 20 min and the supernatant filtered through a 0.2 um Sterivex filter into a 50 ml tube and stored at 4 °C.

#### Viral genome sequencing

Sequencing of Nahant Collection viruses was described in previous work^19^, and was performed as follows. For DNA extraction approximately 18 ml of phage lysate was concentrated using a 30 kD centrifugal filtration device (Millipore, Amicon Ultra Centrifugal Filters, Ultracel 30 K, UFC903024) and washed with 1:100 2216MB to reduce salt concentrations inhibitory to downstream nuclease treatments. Concentrates were brought to approximately 500 ul using 1:100 diluted 2216MB and then treated with DNase I and RNase A (Qiagen RNase A 100 mg mL^−1^) for 65 min at 37 °C to digest unencapsidated nucleic acids. Nuclease treated concentrates were extracted using an SDS, KOAc, phenol-chloroform extraction and resuspended in EB Buffer (Qiagen 19086) for storage at 20 °C. Phage genomic DNA was sheared by sonication in preparation for genome library preparation. DNA concentrations of extracts were determined using PicoGreen (Invitrogen, Quant-iT PicoGreen dsDNA Reagent and Kits P7589) in a 96-well format and samples brought to 5 ug in 100 ul final volume of PCR-grade water diluent for sonication. Samples were sonicated in batches of 6 for 6 cycles of 5 min each, at an interval of 30 s on/off on the Low Intensity setting of the Biogenode Bioruptor to enrich for a fragment size of ~300 bp. Illumina constructs were prepared from sheared DNA as follows: end repair of sheared DNA (NEB, Quick Blunting Kit, E1201L), 0.72×/0.21× dSPRI (AMPure XP SPRI Beads) size selection to enrich for ~300 bp sized fragments, ligation (NEB, Quick Ligation Kit, M2200L) of Illumina adapters and unique pairs of forward and reverse barcodes for each sample, SPRI (AMPure XP SPRI Beads) clean up, nick translation (NEB, Bst DNA polymerase, M0275L), and final SPRI (AMPure XP SPRI Beads) clean up (Rodrigue et al., 2010). Constructs were enriched by PCR using PE primers following qPCR-based normalization of template concentrations. Enrichment PCRs were prepared in octuplicate 25 ul volumes, with the recipe: 1 ul Illumina construct template, 5 ul 5x Phusion polymerase buffer (NEB, 5X Phusion HF Reaction Buffer, B0518S), 0.5 ul 10 mM dNTPs (NEB, dNTP Mix (1 mM; 0.5 ml), N1201AA), 0.25 ul 40 uM IGA-PCR-PE-F primer, 0.25 ul 40 uM IGA-PCR-PE-R primer, 0.25 ul Phusion polymerase (NEB, Phusion High Fidelity DNA Pol, M0530L), 17.75 ul PCR-grade water. PCR thermocycling conditions were as follows: initial denaturation at 98 °C for 20 sec; batch dependent number of cycles (range of 12–28) of 98 °C for 15 sec, 60 °C for 20 see, 72 °C for 20 sec; final annealing at 72 °C for 5 min; hold at 10 °C. For each sample 8 replicate enrichment PCR reactions were pooled and purified by 0.8x SPRI beads (AMPure XP) clean up. Each sample was then checked by Bioanalyzer (2100 expert High Sensitivity DNA Assay) to confirm the presence of a unimodal distribution of fragments with a peak between 350–500 bp. Sequencing of phage genomes was distributed over 4 paired-end sequencing runs as follows: HiSeq library of 18 samples pooled with 18 external samples, 3 MiSeq libraries each containing ~100 multiplexed phage genomes. Accession numbers for all sequenced phage genomes are provided in Supplementary Data 1, where they are identified as phageSet283; the subset of phages used in the majority of analyses in this work are identified as phageSet248 and exclude non-independent isolates derived from the same plaque, as well as well as identical phages isolated from multiple independent plaques from the same host strain in the bait assay.

#### Viral protein clustering

To characterize and annotate groups of proteins in assembled viral genomes in the Nahant Collection^19^, proteins were clustered using MMseqs2 v. 2.23394^69^ with default parameter settings, the 21,937 proteins reported in the GenBank files associated with each of the 283 Nahant Collection phage genomes were clustered into 5,929 clusters including 2,978 singletons. MMseqs2 cluster assignments for each protein sequence are provided in Supplementary Data 6.

#### Viral protein cluster annotation

All proteins were annotated using InterProScan^70^ v.5.39–77.0; eggNOG-mapper^71,72^ v.2 using both automated and viral HMM selection options; Meta-iPVP^73^; and with best matches to 9518 Viral Orthologous Groups^74^ HMM profiles (obtained at http://dmk-brain.ecn.uiowa.edu/pVOGs/downloads.html); search was performed with hmmer, requiring a bitscore of 50 or greater (highest e-value 5.80E-13), as follows: *hmmsearch -o $out_dir/$hmm_group.$hmmfile.$prots_short_name.hmm.out -tblout $out_dir/$hmm_group.$hmmfile.$prots_short_name.hmm.tbl.out -noali -T 50 $hmmfile $prots_dir/$prots_file*. Annotations for viral protein clusters are provided in Supplementary Data 6.

Receptor binding proteins (RBPs) were annotated as follows. RBPs were defined here to include both globular and fibrous host interacting proteins and general protein annotations were reviewed for similarity to known phage receptor binding proteins and supplemented with Phyre2^75^, HHpred, and literature review^76^. Annotated RBPs were mapped onto phage genome diagrams and additional RBPs were annotated based on gene order conservation with phages in the same genus for which RBPs were already identified; annotated RBPs were then used to iteratively search against all Nahant Collection phage proteins using the jackhmmer search tool in the HMMER^77^ v.3.2.1 package (*jackhmmer -cpu 16 -N 3 -E 0.00001 -incE 0.01 -incdomE 0.01 -o $run.$1.vs.$2.jackhmmer.iters-$iters.out -tblout $run.$1.vs.$2.jackhmmer.iters-$iters.tbl.out -domtblout $run.$1.vs.$2.jackhmmer.iters-$iters.dom.tbl.out $queryFASTAS $subjectFASTAS*) and new hits were manually reviewed. All annotations were performed on a protein-cluster level and annotations of proteins and protein clusters as “adsorption - RBP” are indicated in Supplementary Data 6.

Recombinases were annotated as follows: Homologs of single strand annealing protein recombinases in the Rad52, Rad51 and Gp2.5 superfamilies in the Nahant Collection phages were identified as described below. First, iterative HMM searches were performed against the Nahant Collection phage proteins using as seeds 194 recombinases identified in Lopes et al.^44^ (excluding RecET fusion protein YP_512292.1; http://biodev.extra.cea.fr/virfam/table.aspx), these represent 6 families of SSAP recombinases (UvsX, Sak4, Sak, RedB, ERF, and Gp2.5); searches were performed using the jackhmmer function of HMMER v.3.1.2 (*jackhmmer -cpu 16 -N 5 -E 0.00001* -*incE 0.01 -incdomE 0.01 -o $run.$1.vs.$2.jackhmmer.out -tblout $run.$1.vs.$2.jackhmmer.tbl.out -domtblout $run.$1.vs.$2.jackhmmer.dom.tbl.out $queryFASTAS $subjectFASTAS*) – this yielded 156 proteins. Second, all hits were plotted onto genome diagrams for all phages in the collection and additional candidate recombinases identified based on gene neighborhood comparisons (Supplementary Data 9) – this step identified 4 additional protein clusters (mmseqs 297, 149, 2211, and 600), totaling 224 proteins. Third, all proteins clusters were curated by manual review of annotations made using InterProScan^70^, EggNOG-mapper^71^, and Phyre2^75^ (annotations provided in Supplementary Data 6) to identify potential false positives (none identified), and references to recombinases in annotations. Where these annotation methods did not provide additional support, sequences were evaluated for additional support using HHpred^78^ (*hhsearch -cpu 8 -i../results/full.a3m -d /cluster/toolkit/production/databases/hh-suite/mmcif70/pdb70 -o../results/2058109.hhr -oa3m../results/2058109.a3m -p 20 -Z 250 -loc -z 1 -b 1 -B 250 -ssm 2 -sc 1 -seq 1 -dbstrlen 10000 -norealign -maxres 32000 -contxt /cluster/toolkit/production/bioprogs/tools/hh-suite-build-new/data/context_data.crf*) as implemented on the MPI Bioinformatics Toolkit webserver (mmseq 2896 and 5138 both gave >99% probability hits to DNA repair protein Rad52 with PDB ID 5JRB_G), or JackHMMER (-E 1 -domE 1 -incE 0.01 -incdomE 0.03 -mx BLOSUM62 -pextend 0.4 -popen 0.02 -seqdb uniprotkb) as implemented on the EMBL-EBI webserver (mmseq 2990 showed hits to diverse RedB family RecT-like sequences at e-value ≤1e-05). Following this third step, there were 3 protein clusters for which support was limited, these were included in the final dataset as putative SSAP recombinases but are highlighted here. Protein cluster mmseq 297 (present in 21 phages in 6 genera): was always encoded by genes adjacent to genes in protein cluster mmseq 3923, which was itself a recombinase associated exonuclease that was found either adjacent to mmseq 297 or to the well-supported putative SSAP recombinase mmseq 3721 (sometimes separated by one gene from mmseq 3721). Protein cluster mmseq 600 (present in 2 phages in 2 genera): was encoded adjacent to a protein cluster annotated as a recombination associated exonuclease; iterative HHMER searches of a mmseq 600 cluster representative (AUR82881.1) against Viruses in UniProtKB using jackhmmer yielded hits to proteins in mmseq 297 in iteration 3. Protein cluster mmseq 2990 (present in 1 phage): was encoded adjacent to two small proteins encoding putative recombination associated exonucleases and was in the same genomic position relative to neighboring genes as putative recombinases in related phages in the genus. Finally, all putative SSAP recombinase genes were assigned to a recombinase family by clustering based on 2 iterations of all-by-all HMM jackhmmer sequence similarity searches of all candidates and the reference seed set of Lopes^44^ (*jackhmmer -cpu 16 -N 2 -E 0.00001 -incE 0.01 -incdomE 0.01 -o $run.$1.vs.$2.jackhmmer.out -tblout $run.$1.vs.$2.jackhmmer.tbl.out -domtblout $run.$1.vs.$2.jackhmmer.dom.tbl.out $queryFASTAS $subjectFASTAS*); similarities were were visualized using Cytoscape v.3.3.0 using the “Edge-weighted Spring Embedded Layout” based on jackhmmer score, clusters were identified using the ClusterMaker2 v.1.2.1 Cytoscape plugin with the MCL cluster option and all settings at default and Granularity=2.5. Proteins in 3 mmseq clusters (149, 297, 600) did not fall into MCL clusters with recombinases from the annotated seed set and therefore are described as “unknown” rather than being assigned to a family of recombinases. All final assignments of genes to a recombinase superfamily and family, as well as all associated annotations, are provided in Supplementary Data 6 (sheet A.prots_overview column anno_Recombinase_manual). Additional details regarding seed sequences and MCL cluster assignments associated with recombinase analyses are provided in Supplementary Data 7 which contains a main descriptor sheet (00.readme), an overview of the 224 Nahant phages with recombinases (sheet 01.NahantPhageRecombinases_224), a table of InterPro domains associated with each of the reference and Nahant recombinases, with specific mmseqs and MCL clusters (sheet 02.IPR_annos_Lopes+Nahant), a list of all references used (sheet 03.List1_LOPES_ALL.noETfusion), the output of the iterative jackhmmer search with seeds against all Nahant Collection proteins (sheet 04.List1_vs_NahantProts), the output of the all-by-all jackhmmer search for 194 references and 224 putative Nahant recombinases (sheet 05.Lopes+Nahant224_v_self2iter), and information on the assignment of all Nahant and reference proteins to MCL clusters as shown in Fig. 6 (06.Recombinase_assign_by_MCL).

All proteins were assigned to one of three broad categories - structural, other (non-structural), or no prediction - based on manual review of annotations derived from: NCBI product ID, Virfam^21^, PhANNs^79^, pVOGs^74^, eggNOG-mapper^72^, Phyre2^75^, the MPI Bioinformatics implementation of HHpred^78^, and targeted annotations of predicted receptor binding proteins and recombinases (see descriptions for targeted annotations in Methods, above). Protein clusters (mmseq groups) were reviewed for conflicting calls and ultimately all proteins within each protein cluster (mmseqsID) were assigned to a single category. All assignments, and annotations on which they were based, are provided in Supplementary Data 6.

The approach for assigning annotations to these broad categories was as follows: Step 1) All genes identified as putative recombinases through targeted annotations were assigned as “other”. Step 2) All genes identified as putative receptor binding proteins through targeted annotations were assigned as “structural”. Step 3) Genes not assigned to a category in steps 1 and 2, and which were identified by Virfam as “head-neck-tail” associated were assigned as follows: Genes annotated by Virfam as a terminase (TerL) were assigned as “other”; genes annotated by Virfam as a major capsid protein (MCP), portal (portal), adaptor (Ad1, Ad2, Ad3), head-closure (Hc1, Hc2, Hc3), tail completion (Tc1, Tc2), major tail protein (MTP), neck (Ne), or sheath (sheath) were assigned as “structural”. Step 4) Genes not assigned to a category in steps 1–3, were assigned as “structural” or “other” (non-structural) if identified as such by PhANNs with a confidence of ≥95%. Cases where conflicting annotations were observed between PhANNs and other annotations were flagged for review in subsequent steps. Step 5) Genes with annotations of VOG0263 (DNA transfer protein); terminal protein, any reference to internal virion protein, DNA circularization protein, and MuF-like proteins were assigned as “other”; in the case of conflict the Step 5 annotation superseded the prior annotations. Step 6) Genes with annotation as a terminase (large subunit, small subunit, and unspecified) by any of the tools (requiring ≥ 90% confidence if based on Phyre2) were assigned as “other”. Step 7) All genes lacking support across annotations were assigned as “no prediction”, high confidence Phyre2 predictions qualitatively judged as inappropriate were disregarded. Step 8) Genes flagged in Step 4 were reviewed and assigned as “structural” when containing any structural related genes (i.e. those listed in Step 3 and any others identifiable as structural based on words in the annotations and consensus across tools, e.g. containing the word baseplate, capsid, coat, head, spike, tail, whisker, fibritin). Additional targeted annotation by HHpred was used to facilitate assignment to “structural” (known structural proteins as described for Step 3 and in the aforementioned list), “other” (non-structural), “no prediction” (e.g. no assignable function based on available annotations and a PhANNs confidence of <95% for its category of “other”). Step 9) All protein clusters (genes with the same mmseqsID) were reviewed for consistency of annotation among member genes, and additional targeted annotation by HHpred was used to facilitate assignment to “structural” (known structural proteins as described for Step 3 and Step 8), “other” (non-structural), “no prediction” (e.g. no assignable function based on available annotations, a PhANNs confidence of <95% for its category of “other”). In cases where existing assignments of genes within the protein cluster contained both “no prediction” and “other” calls, the “no prediction” call prevailed where these represented more than ~30% of the calls across all genes in the cluster.

### Annotation of viral potential for temperate lifestyle

#### Overview

We identified 6 genera of phages as likely representing temperate phages (indicated in Fig. 2).

#### Bacterial genome read mapping

In order to evaluate the possibility that phages closely related to the Nahant Collection phages reside in the bacterial hosts in this study as prophages we used a read mapping approach. Briefly, reads from each of 276 bacterial genomes isolated from Nahant were mapped against each of the 248 phages and coverage in terms of total bases and genes was considered. Overall, results of this analysis are consistent with our other approaches for assessing potential for lysogeny in these phages. Confirmed as prophages are the known active prophage (1 phage, NCVICG_31; this is the sole case of a prophage being isolated from its own host of isolation and was the sole phage in NCVICG_31, a myovirus in the subfamily *Peduovirinae*) and the transposable phages (7 phages, NCVICG_41), both genera of which show extensive recruitment of bacterial reads, covering 100 and 93% of their genomes, respectively. In addition to these 8 phages, 58 phages recruited bacterial genome reads covering up to 510 bases (range: 30–510 bases covered). Investigation of the genes to which these reads mapped showed that in only one case was a gene covered at ≥ 70%, this was a single strand binding protein in one phage in NVICG_43 (a group which includes 20 members). Where there was any mapping of reads the patterns were bimodal, with either extensive coverage (which we describe in Fig. 2 as strong evidence, in category A) or very limited coverage (which we note in Fig. 2 as weak evidence, in category B).

Results of the analyses are reported in the supplementary data as follows: The total number of bases covered by bacterial genome reads for each individual phage genome are reported in a summary form that indicates both the total bases covered when all bacterial genome reads are considered together (aggregate) and when each bacterial genome’s reads are considered alone (individual) (Supplementary Data 5); additional data on minimum, maximum, and average read depth for each phage and each analysis type (individual vs aggregate) are also reported (Supplementary Data 5); all genes covered at ≥70% by bacterial genome mappings are indicated in the column entitled reads_mapping_from_bacterial_genome in sheet D of Supplementary Data 5.

Read mapping analyses were performed as follows: Demultiplexed bacterial genome reads were quality trimmed using fastp^80^ v.0.20.1 with default settings (example: *fastp -i $1\_1.fastq -I $1\_2.fastq -o $fastpDir/$1\_1.FASTP.fastq -O $fastpDir/$1\_2.FASTP.fastq -html $fastpDir/$1.FASTP.html -json $fastpDir/$1.FASTP.json*). Phage genomes were indexed for read mapping using bwa^81^ v.0.7.17 with default settings example: Forward and reverse reads bacterial genome reads were then independently mapped onto phage genomes and resulting bam files sorted using samtools^82^ v.1.8 (here the example for mapping of forward reads: *while read -r line; do cd $phageDir; (bwa mem -t 12 $line $reads/$1\_1.FASTP.fastq | samtools view -h -F 4 - | samtools sort -@12 -o $interim/$1.vs.$line.BWA_MEM_ALN.FORWARDv2.sam.sorted.bam -) 2» $interim/$1.vs.$line.BWA_MEM_ALN.FORWARDv2.sam.sorted.bam.log; done < $scriptsDir/phageGenomes.names*). Forward and reverse read mappings were merged and sorted using samtools and genome coverage evaluated using bedtools^83^ v.2.29.2; this was done using both an “individual” approach where reads were merged only for each bacterial genome alone versus each phage (example: while read -r line; do cd $interim; (*samtools merge -@$SLURM_CPUS_PER_TASK - $1.vs.$line.BWA_MEM_ALN.FORWARDv2.sam.sorted.bam $1.vs.$line.BWA_MEM_ALN.REVERSEv2.sam.sorted.bam | samtools sort -@$SLURM_CPUS_PER_TASK - -o - | bedtools genomecov -ibam - -d > $1.vs.$line.BWA_MEM_ALN.FandR.merged.sorted.bam.bed) 2» $1.vs.$line.merge2bed.log; done < $scriptsDir/phageGenomes.names* #note that the .bed file is in fact a genomecov output file format and not a bed file format) and using an “aggregate” approach where reads from all bacterial genomes were merged for mapping onto each phage genome (example: while read -r line; do cd $interimSelect276; (samtools merge -@$SLURM_CPUS_PER_TASK - 10 N*.vs.$line.BWA_MEM_ALN.* | samtools sort -@$SLURM_CPUS_PER_TASK - -o $outDir276/ALL276.vs.$line.merged.sorted.bam) 2» $outDir276/ALL276.vs.$line.merge2bam.log; done < $scriptsDir/phageGenomes.names followed by while read -r line; do cd $interimSelect276; (bedtools genomecov -ibam $outDir276/ALL276.vs.$line.merged.sorted.bam -d > $outDir276/ALL276.vs.$line.merged.sorted.bam.genomecov) 2» ALL276.vs.$line.bam2genomecov.log; done < $scriptsDir/phageGenomes.names). Summary mapping information was obtained from genomcov files using awk^84^ (example: *while read -r line; do cd $interim; (awk -v OFS = ‘\t’ ‘BEGIN {count = 0} {if ($3 > 1) count=count+1} END {print FILENAME,count,“coveredBases”}’ $1.vs.$line.BWA_MEM_ALN.FandR.merged.sorted.bam.bed » $1.vs.$line.awkGenomeCovInfos.summary && awk -v OFS = ‘\t’ ‘END {print FILENAME,NR,“totalBases”}’ $1.vs.$line.BWA_MEM_ALN.FandR.merged.sorted.bam.bed » $1.vs.$line.awkGenomeCovInfos.summary && awk -v OFS = ‘\t’ ‘{sum=sum + $3} END {print FILENAME,sum/NR,“averageDepth”}’ $1.vs.$line.BWA_MEM_ALN.FandR.merged.sorted.bam.bed » $1.vs.$line.awkGenomeCovInfos.summary && awk -v OFS = ‘\t’ ‘BEGIN {max=0} {if ($3>max) max = $3} END {print FILENAME,max,“maxDepth”}’ $1.vs.$line.BWA_MEM_ALN.FandR.merged.sorted.bam.bed » $1.vs.$line.awkGenomeCovInfos.summary && awk -v OFS = ‘\t’ ‘BEGIN {min=999999} {if ($3<min) min = $3} END {print FILENAME,min,“minDepth”}’ $1.vs.$line.BWA_MEM_ALN.FandR.merged.sorted.bam.bed » $1.vs.$line.awkGenomeCovInfos.summary && awk -v OFS = ‘\t’ ‘BEGIN {count = 0} {if ($3 = =0) count=count+1} END {print FILENAME,count,“uncoveredBases”}’ $1.vs.$line.BWA_MEM_ALN.FandR.merged.sorted.bam.bed » $1.vs.$line.awkGenomeCovInfos.summary) 2» $1.vs.$line.awkGenomeCovInfos.log; done < $scriptsDir/phageGenomes.names*). Phage genome gff3 files were converted to bed files using bedops^85^ v.2.4.39 (example: for file in *gff3; do cd $gffDir; gff2bed < $file > $file.bed; done) and any genes with 70% or more overlapped by recruited read regions identified using bedops (example: *while read line; do cd $gffDir; bedops -element-of 70% $line.gff3.bed.GENE.bed ALL276.vs.$line.merged.sorted.bam.bed » ALL276.vs.$line.genesCovered_70pct.bed; done < $scriptsDir/phageGenomes.names*). All programs were installed using conda v.4.9.0.

#### Gene-based annotation

Integrases were identified in phages in 5 of the genera. The phages in NCVICG_12 (2 phages), NCVICG_24 (4 phages), and NCVICG_31 (1 phage), were identified as encoding integrases based on annotations with iterative jackhmmer searches with PF00589 phage integrase seed alignment, and by EggNOG-mapper and InterProScan annotation. The phages in NCVICG_23 and NCVICG_28 were identified as encoding PF00239 family integrases based on InterProScan annotation. The phages in NCVICG_41 (7 phages) were identified as encoding a Mu-like transposase, PF02914, on the basis of InterProScan annotations. Iterative jackhmmer searches with N15 phage linear plasmid maintenance gene SopA (NP_046923.1) and *E. coli* ParA (AAA99230.1) yielded no hits to any of the Nahant Collection phages. Finally, phages in 19 genera in the collection encode genes with transcriptional repressor domains represented by IPR010982, IPR010744, IPR001387, IPR032499.

### Viral genome clustering

To understand how the diversity of viral genomes in the Nahant Collection is organized, we use the VICTOR classifier^32^, which determines genome to genome distances between concatenated amino acid sequences of viral proteomes using the Genome-BLAST Distance Phylogeny method^86^ and clusters these using OPTSIL^87^ and criteria optimized by benchmarking to reference ICTV prokaryotic virus taxonomic units available at the time of the development of the tool^32^, with the fraction of links required for cluster fusion of 0.5^88^. Average support values for the phylogenomic trees using the d_0_, d_4_, and d_6_VICTOR formulas were 49%, 31%, and 51%, respectively, and results presented here were those derived from the d_6_ formula, for which 171 species-level and 49 genus-level clusters. VICTOR taxonomy assignments for all phages included in the analysis are provided in Supplementary Data 1 sheet B, where they are identified as phageSet283. Genome diagrams of all phages ordered by VICTOR distance are shown in Supplementary Data 8 (with all genes colored by and labeled based on the protein cluster number to which they belong) and Supplementary Data 9 (with only recombinases highlighted), these figures were generated in R v.3.6.1 with the packages genoPlotR v.0.8.10, ape v.5.4-1, and readr v.1.3.1.

### Viral genome relatedness to previously described phages

To assess the robustness of the predicted VICTOR-based genus level groupings and their relation to previously identified viruses with known hosts we clustered the Nahant Collection phages with previously described phage genomes. We use the curated list of NCBI phage genomes generously provided as a public resource by the laboratory of Andrew Millard (16,103 phage genomes: http://s3.climb.ac.uk/ADM_share/crap/website/26Aug2019_phages.gb.gz; methods now published^89^). We reduced the full list of 16,103 phage genomes to 10,663 genomes based on 95% identity clustering using the dedupe.sh tool in BBtools; we then added Nahant Collection phages (identified as phageSet283 in Supplementary Data 1 sheet B) not already included in the list, yielding a total of 10,722 phage genomes (see Supplementary Data 4 sheet A for a list of all phage genome accessions included). We next used vConTACT2 v.0.9.10^29^ to predict viral genome clusters (*vcontact -raw-proteins $rawprots -rel-mode ‘Diamond’ -proteins-fp $gene2genome -db ‘ProkaryoticViralRefSeq94-Merged’ -pcs-mode MCL -vcs-mode ClusterONE -c1-bin /home/k6logc/miniconda3/bin/cluster_one-1.0.jar -output-dir $outdir*; see Supplementary Data 4 sheet B for full clustering output for all members).

The vConTACT2 analysis allowed us to identify 47 previously described phages that belong to 17 Nahant Collection VICTOR genera; none of these were found to be members of the same species as Nahant phages when examined together with VICTOR (Supplementary Data 4 for information about 47 previously described phages). The majority of previously described phages in Nahant Collection VICTOR genera also infected hosts in either the *Vibrionaceae* or *Shewanellaceae* (a second family of hosts also represented in this study), consistent with a previous finding that phage genera are specific to host families^32^. However, in 4 of the 17 genera, previously described phages had hosts in Gammaproteobacterial orders that were non-*Vibrionales*, including the *Enterobacterales*, *Aeromonadales*, *Pasteurellales*, and *Alteromonadales*. The genus of phages for which previous isolates had the most diverse hosts (NCVICG_31) contains phages that, in the 2019 taxonomy revision by the International Committee on the Taxonomy of Viruses, were assigned to multiple genera within the phage subfamily *Peduovirinae*; and was represented in the Nahant Collection by only a single phage - the sole case in this study of isolating a host-derived prophage (as the result of a prophage forming a plaque on its own host in the bait assay).

With respect to the Nahant phages alone, correspondence between VICTOR genera and vConTACT2 clusters was overall high (see Supplementary Data 4 sheet C for cluster assignments for Nahant phages and Supplementary Data 4 sheet D for correspondence between genera and vConTACT2 clusters). However, we found that a number of VICTOR genera (NCVICG) over-clustered phages as compared with vConTACT2, as follows. The representatives of the non-tailed family *Autolykiviridae* were identified as all being members of a single genus by VICTOR (NCVICG_17) but were split into 2 vConTACT2 clusters. Representatives of NVCG_36 were split across 2 vConTACT2 clusters. The two phages in NCVICG_36 were identified as separate outliers in vConTACT2. The phages in NCVICG_47 were split across 3 vConTACT2 subclusters within a single cluster. And, three VICTOR genera (NCVICG: 14, 21, and 46) that otherwise exactly corresponded with vConTACT2 clusters, each also included a member classified as an outlier by vConTACT2. Finally, 7 phages (members of NCVICG: 7, 9, 12, and 28) that were included as inputs to the vConTACT2 analysis were excluded from the summary outputs in multiple run attempts for reasons that are unclear.

### Host range

#### Host range assay

Host range of viruses was determined as follows, and as also previously described^13,20^. Cell-free phage lysates were stamped onto host agar overlay lawns and observed for changes in lawn morphology proximal to each stamp. Phage application to host lawns was performed using a 96-well blotter (BelArt, Bel-blotter 96-tip replicator 378760002) that was set into a microtiter plate containing arrayed phage lysate, transferred to the surface of the host lawn, and allowed to remain in contact for several minutes. Each 96-stamp contained 3 replicates of each phage lysate, distributed across three panels (columns 1–4, 5–8, 9–12) each with a unique array of the 32 samples (including one negative control). 96-well blotters were microwave steam sterilized (Tommee Tippee, Closer to Nature Microwave Steam Sterilizer) in batches for continuing re-use during plating sessions. Bacterial strains were prepared for the infection assay by inoculating 1 ml volumes of 2216MB in 2 ml 96-well culture blocks directly from glycerol stocks and shaking them at RT for approximately 48 h. Agar overlays were prepared by transferring 250 ul aliquots of host culture to bottom agar plates (2216MB, 1% Bacto Agar, 5% glycerol; in 150 mm diameter plates) and adding 7 ml of molten 52 °C top agar (2216MB, 0.4% Bacto Agar, 5% glycerol). Phages were prepared by distributing lysates into a 2 ml 96-well culture block in panels as described above, aliquots of <200 ul were then transferred into shallow microtiter plates so that the blotter could phage lysate by capillary action. Host lawns were stamped with phage lysates within 5–6 h of plating lawns. Agar overlays were assessed for changes in lawn morphology associated with phage treatment and scored blind with respect to phage identity and arrangement of replicates. Plates were scored for the presence of interactions on days 1, 2, 3, 7, 14, 21, and 30, and the outer bands of the interaction zones were marked with a different color for each time point. After 30 days the interactions for each strain were recorded and the approximate diameter for each interaction at each time point was recorded. During recording of the interactions for each plate an additional qualitative measure of confidence in the projected positive or negative call of the interaction was made. For example, where 2 of 3 replicates were positive for a phage on a lawn with no other positive interactions such an interaction would be called by the qualitative measure as “real”; alternatively, where 2 of 3 replicates were positive for a phage on a lawn containing several other positive interactions the qualitative measure might call these replicates “contam” if they were high-titer interactions and occurred in close proximity to other positive interactions. Evaluation of changes in clearing sizes showed that the majority of clearings (>90%) increased in size over the course of the observation period, consistent with these representing replicative infections rather than inhibition or killing from without. If any of the clearings that do not increase in size represent non-replicative interactions this would reduce the total true killing interactions further and underscore our finding of the general sparsity of interactions. A list of all infection pairs and information regarding plaque sizes and increases are provided in Supplementary Data 1 sheet C, and represent interactions between phages identified in the Supplement as phageSet248 and hosts identified as baxSet279.

### Characterization of phage-host interaction features

#### BiMat modularity analysis

To characterize large scale features of the infection network we use the BiMat MatLab package^22^ as described in^5,6,90^. Modularity was quantified using the leading eigenvector method, with a Kernighan-Lin tuning step performed after module detection, and nestedness was quantified using overlap and decreasing fill (NODF). Statistical significance of modularity and nestedness was tested against 1000 random matrices generated using the equiprobable method, preserving the overall matrix connectivity. Modularity values were as follows: Qb value 0.7306, mean 0.4362, std 0.0047, z-score 63.2774, t-score 2001.0077, percentile 100; Qr value 0.9318, mean 0.1004, std 0.0219, z-score 37.9184, t-score 1199.0848, percentile 100. Nestedness values were as follows: Nestedness value: 0.0300, mean 0.0230, std 0.0005, z-score 14.0305, t-score 443.6833, percentile 100. The 248 phages included in the analysis (phageSet248 in Supplementary Data 1) are genome sequenced phages isolated during the Nahant study, excluding cases of duplicate phages purified from the same plaque and excluding duplicate phages purified from different plaques on the same host; the 279 bacteria included in the analyses (baxSet279 in Supplementary Data 1) include all bacterial strains screened in the host range assay for which there was a positive interaction with a phage in phageSet248 (ie. host strains that were assayed but not killed by any phages were not included); these represent 1,436 infections out of a possible set of 69,192 and yield a connectance or fill of 0.021. To facilitate visual comparisons between the matrix of interactions between phages and bacteria with known species assignments (Fig. 2b) and the BiMat results, the representation of the BiMat analysis as shown in the main text (Fig.2a) includes only the subset of interactions between phages in phageSet248 and the 259 bacterial strains (baxSet259) that is the intersection of the 279 bacterial strains (baxSet279) included in the BiMat analysis and the 294 bacterial genomes (baxSet294) for which genomes were available. BiMat module assignments for all phages and hosts are provided in Supplementary Data 1 sheet C.

#### Average nucleotide identity

FastANI v.1.32 was used to determine average nucleotide identity (ANI) for phages and hosts as follows. For phages, run parameters were: kmer size of 16, fragment length of 100, minimum fraction of shorter genome coverage of 75% (*fastANI -kmer 16 -fragLen 100 -minFraction 0.75 -matrix -ql $genomesPathsList -rl $genomesPathsList -o phageSet248_k16fL100Frax75.fastani.out*). For bacteria, run parameters were: kmer size of 16, fragment length of 3000, minimum fraction of shorter genome coverage of 50% (*fastANI -kmer 16 -fragLen 3000 -minFraction 0.5 -matrix -ql $genomesPathsList -rl $genomesPathsList -o baxSet294_k16fL3000Frax50.fastani.out*). Results of ANI analyses are provided in Supplementary Data 1.

#### Host range divergence analyses within VIC-species and VIC-genera

To quantify overlap in host range profiles between phages we develop a metric of host range divergence (represented as concordance (1-divergence) in the main text and Fig. 4). We normalize the binary vector ${{{{{{\boldsymbol{x}}}}}}}_{i}=\left({x}_{1},{x}_{2},\ldots ,{x}_{m}\right)$ representing the killing host range of a phage *i* across all *m* hosts so that it sums to 1, and interpret the result ${p}_{i}={x}_{i}/{\sum }_{j}^{m}{x}_{j}$ as a probability distribution of killing across all *m* hosts for a single phage *i*. We then define the *scaled host range divergence* of a given genus consisting of *n* phages to be the normalized generalized Jensen-Shannon divergence (gJSD) of their infection probability vectors, ${D}_{h}={{\mbox{gJSD}}}\left({{{{{{\boldsymbol{p}}}}}}}_{{{{{{\boldsymbol{1}}}}}}},\ldots ,{{{{{{\boldsymbol{p}}}}}}}_{{{{{{\boldsymbol{n}}}}}}}\right)/{{\log }}_{2}\left(n\right)$. This has the property that $0\le {D}_{h}\le 1$, where ${D}_{h}=0$ means the host ranges of all phages overlap exactly, and ${D}_{h}=1$ means none of the host ranges have any overlap with each other. We note that we took a conservative approach in determining genus-level concordances as presented in Fig. 3 by including all phages within each VIC-genus in the calculation of concordance rather than collapsing intra-species blooms, which are largely comprised of phages with overlapping host ranges. Because high overlap of host range within VIC-species increases the value of the overall genus-level concordance metric (because of the higher number of pairwise comparisons with high values of concordance within species), this approach will be affected by the evenness of species abundances within a genus and including all members yields up to >24 times higher values of the concordance metric than when only including single representatives of each species in this dataset. Calculations of concordance are provided in Source Data Fig. 4 for VIC-genera using both the approach of using all members (Source Data Fig. 4 sheet B, with phages identified as phageSet248) and that of using only single representatives from each VIC-species (Source Data Fig. 4 sheet C, with phages identified as phageSet171); values for VIC-species are provided in Source Data Fig. 4 sheet A. A Welch’s t-test between the set of VIC-genus level concordances and the set of VIC-species level concordances yielded a p-value of 1.45e-07, suggesting that concordance differs significantly between the VIC-genus and VIC-species levels. Analyses and visualizations were performed in R 3.6.1 with the following packages: ggrepel 0.8.1, ape 5.3, combinat 0.0-8, Infotheo 1.2.0, philentropy 0.4.0.9000, igraph 1.2.4.1, ggraph 2.0.0, cowplot 1.0.0, data.table 1.12.2, ggplot2 3.2.1, tidyverse 1.3.0, ggtree 2.0.4, and patchwork 1.1.1.

### Characterization of sequence sharing

#### Homologous recombination within species and genera

To assess the extent of recombination between closely related viruses we used viral species and genera as operationally defined by VICTOR as the framework and estimated effective relative contribution of recombination over mutation (r/m) as follows. HomBlocks v.1.0^91^ was used with default parameter settings to identify, extract, and trim conserved regions within genera based on alignments with progressiveMauve (build February 13, 2015)^92^ and trimAl v.1.2^93^. Phylogenetic relationships between sequences were predicted using IQ-TREE v.1.6.12^94^. ClonalFrameML v.1.12^95^ was then used to evaluate evidence for recombination within species and genera. Estimates of relative effect of recombination to mutation (r/m) were based on the formula r/m = R/theta * delta * nu; where R/theta is the ratio of recombination to mutation rate, delta is mean import length, and nu is the nucleotide distance between imported sequences. We include in our analysis a control set of closely related siphovirus genomes which in a previous study were found to have an r/m of 23.50^35^, accession numbers for these phages are provided in Source Data Fig. 4 sheet G; using the methods we describe here we find a similar r/m of 18.3. All analyses were performed only where there were ≥3 phages per species or genus. For genus level calculations of r/m we performed analyses using two different sets of genomes: first, as presented in Fig. 4e, g including all phages in each genus (Source Data Fig. 4 sheet E, using phages identified as phageSet248); second, including only 1 representative from each species (Source Data Fig. 4 sheet F, using phages identified as phageSet171). As for estimates of host range divergence, estimates of r/m are affected by evenness of species abundances within a genus and including all members can result in reduced estimates of r/m; for example, for NCVICG_17, the *Autolykiviridae*, estimates of genus-level r/m are >285 higher when considering only species representatives rather than all members of the genus.

#### Sharing of 25-mers

To assess nucleotide sequence sharing between phages in the collection overall, we used methods that were not limited by requirement for large scale pairwise genome conservation. We use Mash v.2.2.2^96^ to create a sketch file for all phage genomes (*mash sketch -o $out -k 400000 -s 25 -i $concatGenomesFile*), using a k-mer size of 25 and a sufficiently large sketch size to capture all 25-mers in the largest phage genome; we next generate the mash distance output (*mash dist $out.msh $out.msh -i >> $out.msh.ALLbyALL.dist*), which includes a count of shared 25-mers between all pairs of phage genomes. To compare the infection and k-mer sharing networks, we created a binary vector reflecting whether each pair of phages shares at least one host in the observed infection matrix. Similarly, we created a binary vector reflecting whether each pair of phages shares at least one 25-mer between their respective genomes, as reported by Mash. We then calculated the mutual information between these vectors using the R function mutinformation() from package infotheo 1.2.0. Using mutual information analysis to ask whether phages that share ≥1 25-mer also share ≥1 hosts, we found that one matrix does not predict the other (0.018 bits out of a maximum value of 1 predicted). Host sharing information and Mash outputs for all phage-phage pairs are provided in Source Data Fig. 5 and were based on analysis of phages identified in Supplementary Data 1 as phageSet248.

#### BRIG plotting

To visualize regions of genome conservation and divergence within sets of phages as compared to a reference, as shown in Fig. 4d we used the BLAST Ring Image Generator tool BRIG^97^.

#### Transfers with identifiable directionality between genera

To use a method that could predict directionality of transfer between phages in different genera, we used MetaCHIP^41^, providing VICTOR genus as the grouping variable. This method is a conservative estimator as it requires that candidate regions be conserved within donor groups but not in recipient groups. Modifications were performed to the source code to accept the amino acid-based phylogenetic tree as output by VICTOR as the phylogeny used in the MetaCHIP analysis. MetaCHIP parameters were set to 90% identity cutoff, 200 bp alignment length cutoff, 75% coverage cutoff, 80% end match identity cutoff, and 10 Kbp flanking region length. MetaCHIP results are provided in Source Data Fig. 5.

#### Potential for cross-recruitment of reads between viral genomes

To assess the potential for cross-recruitment of metagenomic reads to lead to false positive detection of phage genomes in metagenomic sequencing datasets we simulated Illumina reads for each of the phage genomes using parameters representative of high quality viral metagenome datasets and then asked whether they yielded a positive identification using a recently developed reference mapping tool designed for rapid characterization of large phage metagenomes. We included 248 phages of the Nahant Collection, as well as 47 previously described phage genomes that in vConTACT2 analyses (above) were found to be members of the same VIC-genera as the Nahant Collection phages. Simulated Illumina paired end reads were generated using DWGSIM v.1.1.11^98^, with settings of: outer distance 500, standard deviation of read lengths 50, number of reads 100000, read lengths for both reads of 250 bp, and error rate 0.0010 (*dwgsim -d 500 -s 50 -N 100000 -1 250 -2 250 -r 0.0010 -c 0 -S 2 $genome $genome.simreads*). Cross recruitment of reads to each of the 295 phages was performed independently for simulated reads from each phage using default settings of FastViromeExplorer^43^ for criteria required to define a genome as present in a read dataset: genome coverage [0.1]; ratio of coverage to expected coverage [0.3], which considers evenness of read mapping; and minimum number of mapped reads [10]. Notably, performing this analysis with 100bp-length read sets eliminated cross-genus mappings that were identified using 250 bp read sets. This is thought to occur because FastViromeExplorer uses a Kallisto^99^ based pseudoalignment approach, which requires only a single matching 31-mer to map a given read to a target genomes, and thus where a 100 bp read and a 250 bp read both share only a single 31-mer with a reference genome the 250 bp read will result in overall greater coverage of the genome. All information about the phages included in the cross-recruitment analysis, and the associated data, are provided in Source Data Supplementary Fig. 7.

### Biological materials availability

Phage and bacterial isolates are available from the authors upon request.

### Reporting summary

Further information on research design is available in the Nature Research Reporting Summary linked to this article.

## Supplementary information

Description of Additional Supplementary Files

Supplementary Information

Supplementary Data 1

Supplementary Data 2

Supplementary Data 3

Supplementary Data 4

Supplementary Data 5

Supplementary Data 6

Supplementary Data 7

Supplementary Data 8

Supplementary Data 9

Reporting Summary

## Data Availability

Information about bacteria and phage isolates, infections, and BiMat module assignments are provided in Supplementary Data 1. Summary data describing host taxa by phage taxa and morphotypes are provided in Supplementary Data 2. Information on assignments of phages to VIRIDIC groups are in Supplementary Data 3. Results of vConTACT analyses are available in Supplementary Data 4, this includes a list of phages obtained from the group website of Andrew Millard (http://s3.climb.ac.uk/ADM_share/crap/website/26Aug2019_phages.gb.gz). Summary data describing mapping of bacterial genome reads to phage genomes are provided in Supplementary Data 5. Annotations of all proteins and protein clusters of phages in this work are provided in Supplementary Data 6. Information regarding annotation phage recombinases is provided in Supplementary Data 7, this includes a table describing seed sequences based on the original source information at http://biodev.extra.cea.fr/virfam/table.aspx. The full VICTOR tree for all phages, together with genome diagrams colored and labeled by protein cluster identifiers, is shown in Supplementary Data 8. The full VICTOR tree for all phages, with recombinases highlighted and colored by type, is shown in Supplementary Data 9. Source data are provided with this paper in the Source Data file for the underlying matrices in Fig. 2, the underlying killing concordance and recombination plots in Fig. 4, underlying host and nucleotide sharing in Fig. 5, and the cross mapping information in Supplementary Fig. 7. New bacterial genomes and *hsp60* sequence data deposited with this work are included under the Nahant Collection of NCBI BioProject with accession number PRJNA328102. Source data are provided with this paper.

## Source data

Source Data

## Code availability

All custom codes associated with this work are available from the authors upon request.
