# Supplementary material for: Resolving the structure of phage–bacteria interactions in the context of natural diversity
Source: Nat Commun. 2022 Jan 18;13:372. doi: 10.1038/s41467-021-27583-z (PMC8766483; doi:10.1038/s41467-021-27583-z)
Supplement: Supplementary file 1 — Description of Additional Supplementary Files [file 41467_2021_27583_MOESM1_ESM.pdf]

Description of Additional Supplementary Files

File Name: Supplementary Data 1
Description: Contains information about bacteria and phage isolates, infections, and BiMat module assignments.

File Name: Supplementary Data 2
Description: Contains information on patterns of host taxon infections with respect to phage taxa and morphotypes.

File Name: Supplementary Data 3
Description: Contains information on correspondence between VIRIDIC and VICTOR phage assignments.

File Name: Supplementary Data 4
Description: Contains results of vConTACT2 analyses.

File Name: Supplementary Data 5
Description: Contains summary data on phage group life history strategy features and mapping of bacterial genome reads to phage genomes.

File Name: Supplementary Data 6
Description: Contains annotation of proteins encoded in phage genomes.

File Name: Supplementary Data 7
Description: Contains information regarding annotation of putative phage recombinases.

File Name: Supplementary Data 8
Description: **Nahant phage genomes in light of VICTOR phylogeny.** VICTOR phylogeny of Nahant Collection phages, with associated genome diagrams annotated with protein cluster identifiers. Phylogenetic tree of phages based on genome BLAST distances of concatenated protein sequences of predicted open reading frames, generated using the VICTOR tool with the d6 clustering formula to define taxa. Bootstraps: values indicated on branches. Tree scale bar (in red): branch lengths represent intergenomic distances scaled in terms of the GBDP distance formula d6, note this was added manually post-plotting based on VICTOR output as GenoPlotR does not include the option to print the tree scale bar. Branch labels indicate: the predicted viral morphotype (auto, sipho, myo, or podo); the phage name (e.g. "1 008 O" for 1.008.O); ordinal day of isolation (222, 261, or 286); the size fraction from which the host of isolation was recovered ("0 2" for 0.2um, "1", "5", and "63"); and the VICTOR taxon cluster (S, species; G, genus; U, subfamily; F, family), note that VICTOR subfamily and family designations are included only for completeness but are generally not supported. Predicted protein coding genes in genome diagrams indicated by block arrows and randomly colored based on protein sequence cluster identity (MMseqs2 ID) to facilitate qualitative inspection of similarities; note that whereas there are 5,929 protein clusters only ~600 colors were used and thus the MMseqs2 ID number indicated in the label with each block arrow must also be considered in evaluating similarities. Underlying annotations are provided in Supplementary Data 6; underlying tree data, in Newick format, provided in Source Data Fig. 2.

File Name: Supplementary Data 9
Description: **Nahant phage genomes in light of VICTOR phylogeny, with predicted recombinase genes highlighted.** VICTOR phylogeny of Nahant Collection phages, with associated genome diagrams annotated with protein cluster identifiers. Phylogenetic tree of phages based on genome BLAST distances of concatenated protein sequences of predicted open reading frames, generated using the VICTOR tool with the d6 clustering formula to define taxa. Bootstraps: values indicated on branches. Tree scale bar (in red): branch lengths represent intergenomic distances scaled in terms of the GBDP distance formula d6, note this was added manually post-plotting based on VICTOR output as GenoPlotR does not include the option to print the tree scale bar. Branch labels indicate: the predicted viral morphotype (auto, sipho, myo, podo); the phage name (e.g. "1 008 O" for 1.008.O); ordinal day of isolation (222, 261, or 286); the size fraction from which the host of isolation was recovered ("0 2" for 0.2um, "1", "5", and "63"); and the VICTOR taxon cluster (S, species; G, genus; U, subfamily; F, family), note that VICTOR subfamily and family designations are included only for completeness but are generally not supported. Predicted recombinase genes indicated by block arrows and colored by predicted recombinase family (ERF: light blue, Redβ: green, Sak: purple, UvsX: light pink; Sak4: dark pink; Gp2.5: yellow; unknown: black). Underlying tree data, in Newick format, provided in Source Data Fig. 2.
