# Supplementary material for: Resolving the structure of phage–bacteria interactions in the context of natural diversity
Source: Nat Commun. 2022 Jan 18;13:372. doi: 10.1038/s41467-021-27583-z (PMC8766483; doi:10.1038/s41467-021-27583-z)
Supplement: Supplementary file 2 — Supplementary Information [file 41467_2021_27583_MOESM2_ESM.pdf]

Supplementary Information

Contents

List of primers used in this work 1

Supplementary figures 2

List of primers used in this work

Primer Name	Primer Sequence (5’ to 3’)	Reference
hsp60-F (H279)	5'-GAA TTC GAI III GCI GGI GAY GGI ACI ACI AC-3'	Goh, S. H. et al. HSP60 gene sequences as universal targets for microbial species identification: studies with coagulase-negative staphylococci. J. Clin. Microbiol. 34, 818–823 (1996).
hsp60-R (H280)	5'-CGC GGG ATC CYK IYK ITC ICC RAA ICC IGG IGC YTT-3'	Goh, S. H. et al. HSP60 gene sequences as universal targets for microbial species identification: studies with coagulase-negative staphylococci. J. Clin. Microbiol. 34, 818–823 (1996).

Supplementary Table 1. Primers used in this study.

Supplementary figures

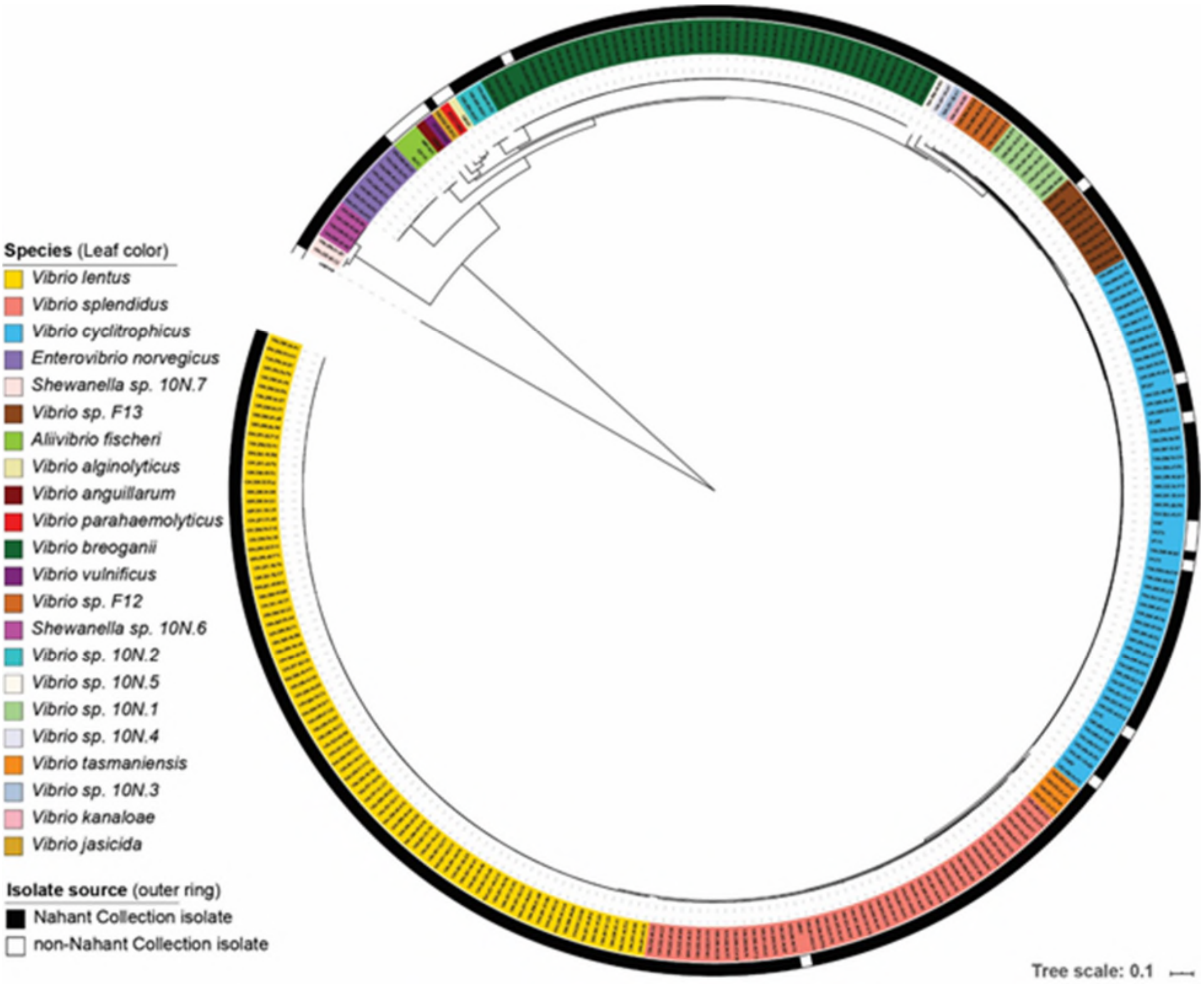

Supplementary Figure 1. Bacterial isolates included in the large scale host range assay include >45 closely related members of each of 4 *Vibrio* species, as well as representatives of at least 18 other species in the family Vibrionaceae. Tree based on concatenated single copy ribosomal protein genes. Underlying bacterial strain information provided in Supplementary Data 1.

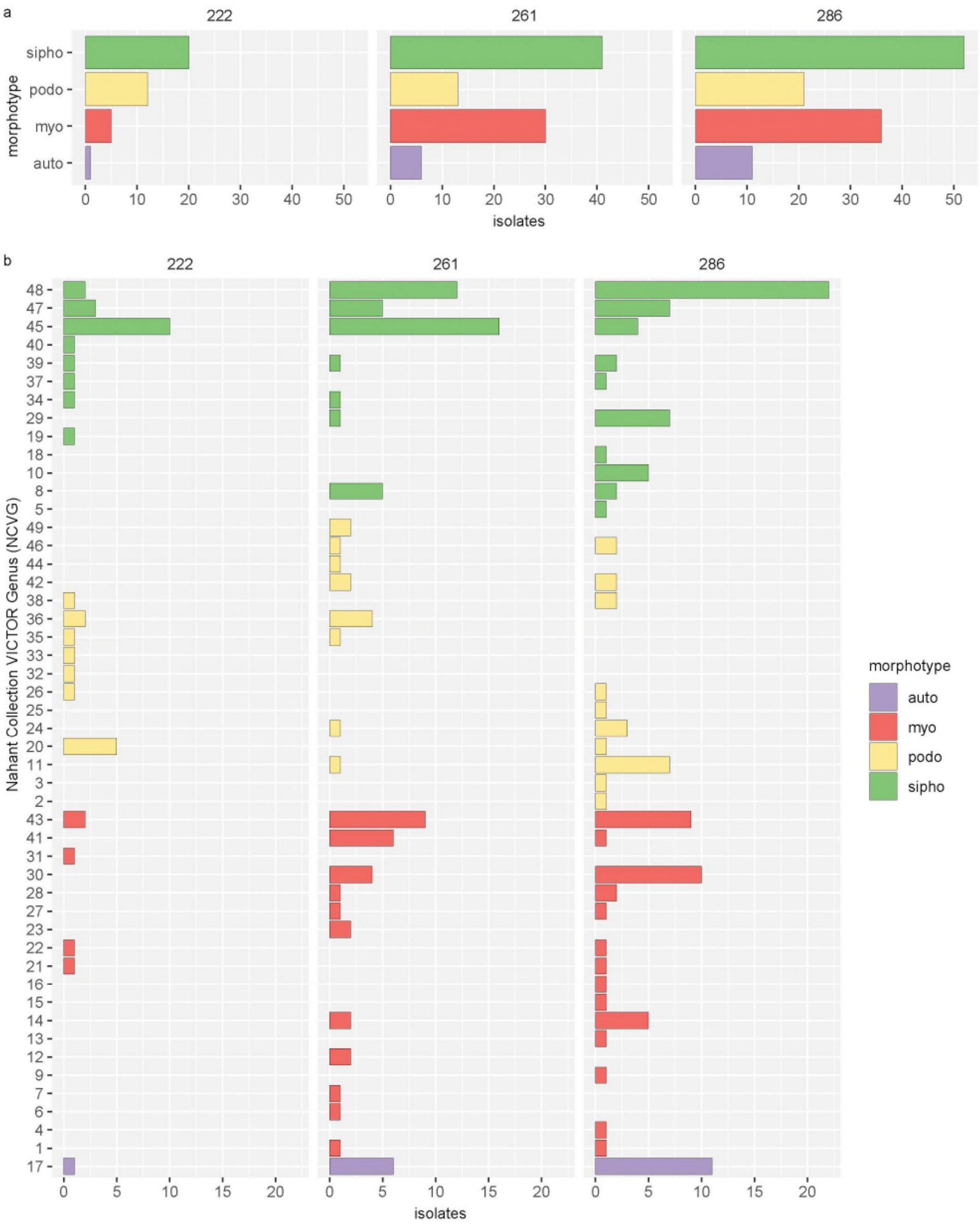

Supplementary Figure 2. Morphotypes and genera of phages isolated on each of the three sampling days. Underlying phage strain information available in Supplementary Data 1.

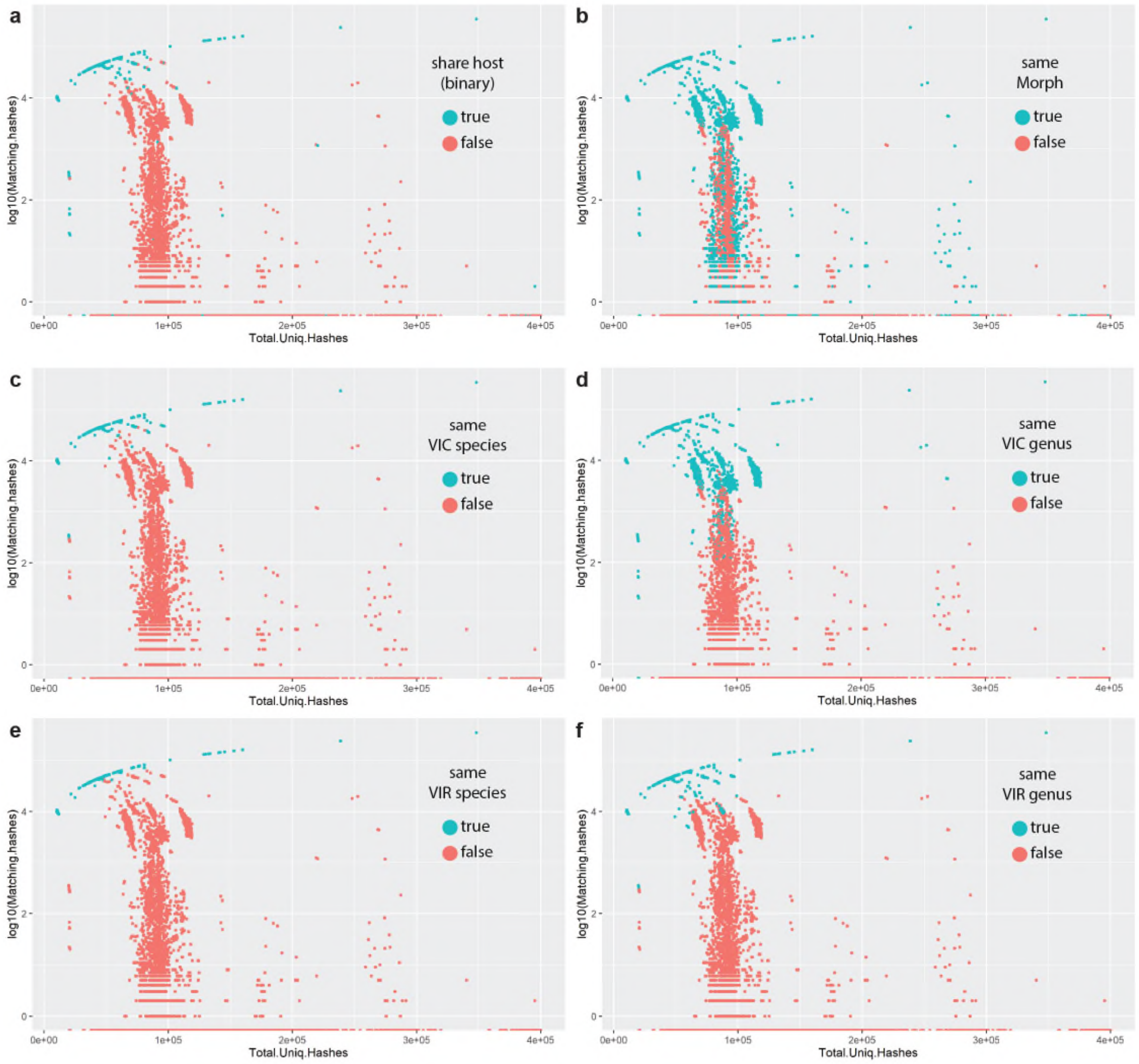

Supplementary Figure 3. Genome similarity in terms of shared 25-mers in light of host sharing, morphology, and operational classification schemes. In all panels **a-f**: the x-axis represents the total number of unique hashes (25-mers) altogether in a pair of phage genomes, the y-axis represents the total number of shared hashes (25-mers) between a pair of phage genomes. All points plotted with jitter, adding random x/y error to spread out overlapping points to facilitate visualization; log(0) values plotted as <0 on the y-axis. Note that the trace of the values representing the highest Y for each X would represent a 1:1 on a linear plot. VIC indicates VICTOR taxa, VIR indicates VIRIDIC taxa. Underlying data available in Source Data Fig. 5.

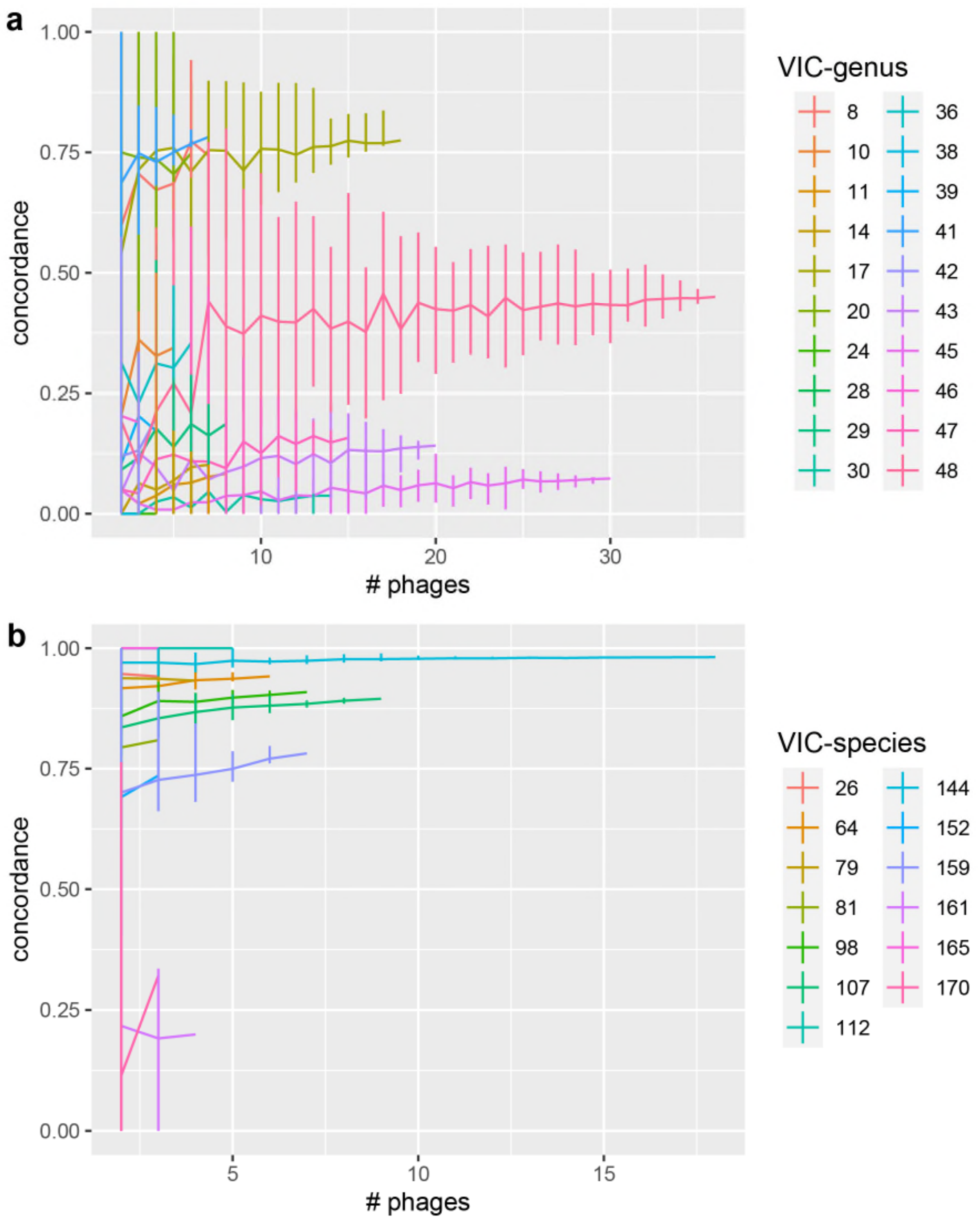

Supplementary Figure 4. Scaling of concordance with size of subsampled groups of phages from VICTOR taxonomic groups of >= 3 phages. Curves represent mean concordance for each # of phages. Vertical lines represent range between maximum and minimum subsampled concordances. **a.** Scaling for VIC-genera. **b.** Scaling for VIC-species.

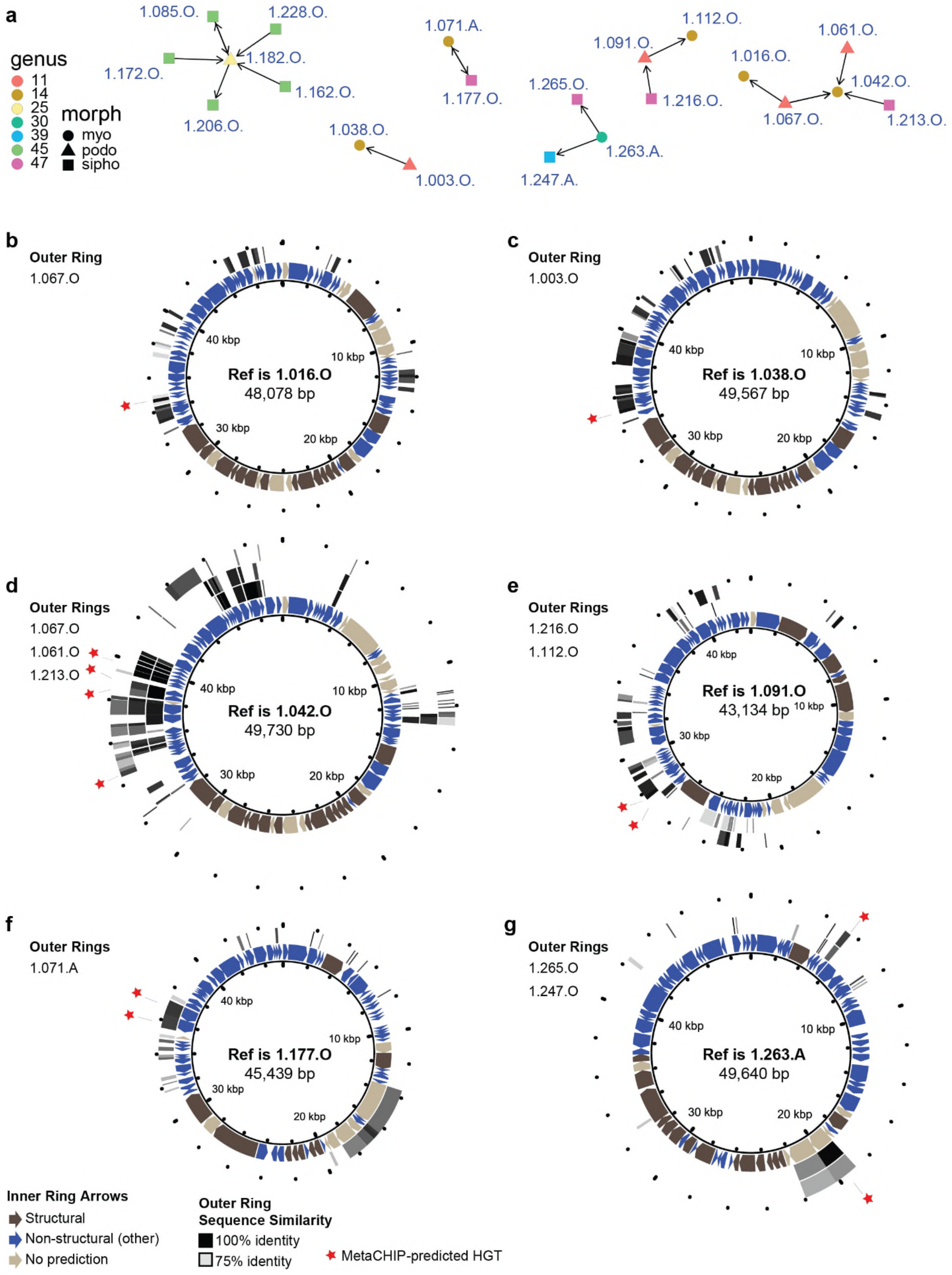
a
genus
11
14
25
30
39
45
47
morph
myo
podo
sipho
1.085.O.
1.228.O.
1.182.O.
1.172.O.
1.162.O.
1.206.O.
1.071.A.
1.177.O.
1.038.O.
1.003.O.
1.265.O.
1.263.A.
1.247.A.
1.091.O.
1.112.O.
1.216.O.
1.061.O.
1.016.O.
1.042.O.
1.067.O.
1.213.O.
b
Outer Ring
1.067.O
Ref is 1.016.O
48,078 bp
c
Outer Ring
1.003.O
Ref is 1.038.O
49,567 bp
d
Outer Rings
1.067.O
1.061.O
1.213.O
Ref is 1.042.O
49,730 bp
e
Outer Rings
1.216.O
1.112.O
Ref is 1.091.O
43,134 bp
f
Outer Rings
1.071.A
Ref is 1.177.O
45,439 bp
g
Outer Rings
1.265.O
1.247.O
Ref is 1.263.A
49,640 bp
10 kbp
20 kbp
30 kbp
40 kbp
Inner Ring Arrows
Structural
Non-structural (other)
No prediction
Outer Ring
Sequence Similarity
100% identity
75% identity
MetaCHIP-predicted HGT

Supplementary Figure 5. Horizontal gene transfer between phages in different genera and of different morphotypes and with no shared hosts killed. a, Candidate gene transfer events between phages in different genera and with different morphotypes were identified by MetaCHIP, which considers conservation with donor groups to predict transfers into recipients. **b-f**, Extent of sequence sharing between pairs identified using MetaCHIP was further examined using BLAST-based mapping using Blast Ring Generator (BRIG), revealing that there were commonly additional regions of high sequence identity in neighboring genomic regions. Predicted open reading frames in phage genomes are depicted as arrows and colored according to broad gene functional categories (see Methods for approach used to make broad functional assignments); stars indicate sequence sites involved in transfer events predicted by MetaCHIP. Underlying data is provided in Source Data Fig. 5 and Supplementary Data 7.

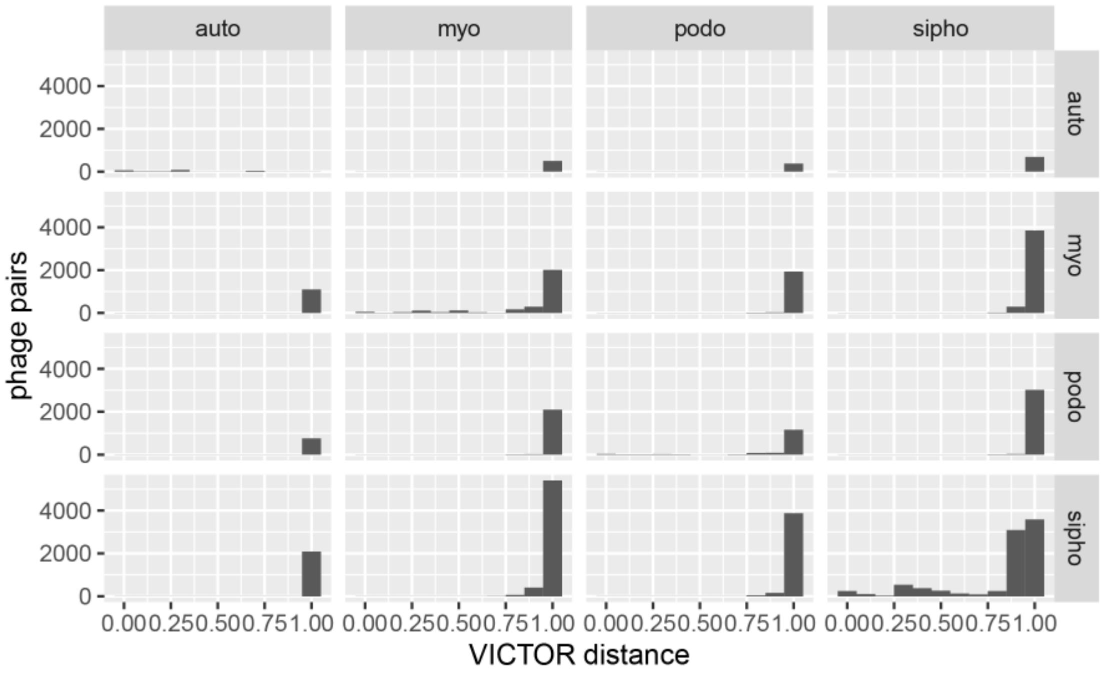

Supplementary Figure 6. Distributions of VICTOR distances between phages of each morphotype. Histograms of genomic distances as calculated by VICTOR between pairs of phages of one morphology (rows) and of the same or a different morphology (columns).

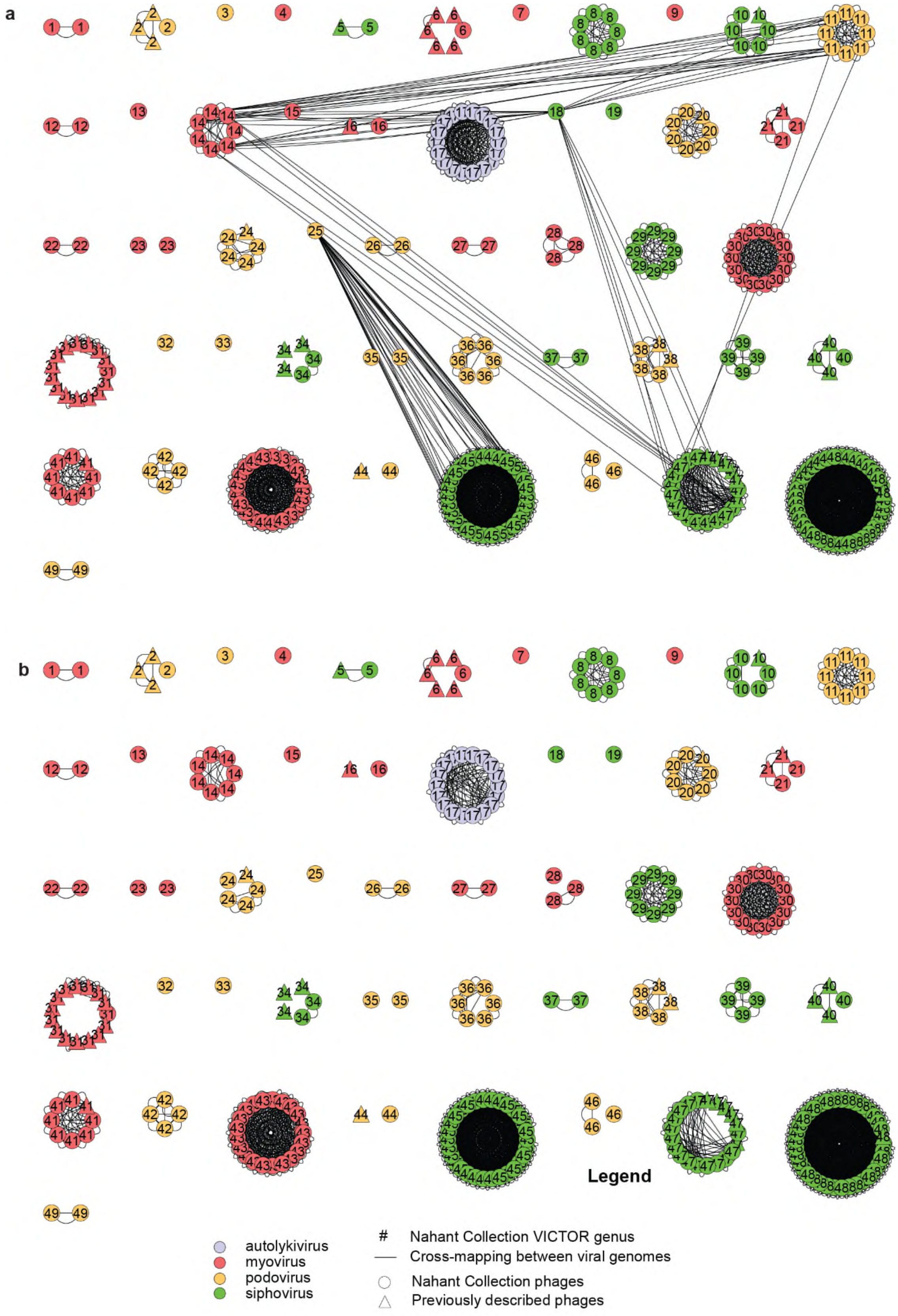
a
b
Legend
autolykivirus
myovirus
podovirus
siphovirus
Nahant Collection VICTOR genus
Cross-mapping between viral genomes
Nahant Collection phages
Previously described phages

Supplementary Figure 7. Non-specific recruitment of reads can lead to false positives in mapping of viral reads to reference genomes. Pseudoalignment-based mapping of reads to reference genomes leads to extensive cross-mapping within genera as well as between phages of different morphotypes (plots ordered by genus, as reflected in labels). Cross-mapping is defined operationally as determination of a virus as “present” by the pseudoalignment-based viral metagenome characterization tool FastViromeExplorer when using default settings and 100,000 simulated reads of either **a**, 250bp or **b**, 100bp. Simulated reads from each virus were tested for mapping against all other phages in the dataset individually, including 248 Nahant Collection phages and 47 previously isolated phages identified as members of Nahant Collection genera. As a result of the requirement for mapping of only a single 31-mer to assign a read as mapped, false-positives are more frequently observed when using longer reads as a result of higher overall coverage. Patterns of cross-mapping vary across genera and range from observations of no within-genus cross-mapping to cases of extensive between-genus cross-mapping, including between genera with phages of different morphotypes. Underlying data, including proportion of reads mapped in comparison with mapping to self, are provided in Source Data Extended Data Fig. 7.
