# Supplementary material for: Resolving the structure of phage–bacteria interactions in the context of natural diversity
Source: Nat Commun. 2022 Jan 18;13:372. doi: 10.1038/s41467-021-27583-z (PMC8766483; doi:10.1038/s41467-021-27583-z)
Supplement: Supplementary file 12 — Reporting Summary [file 41467_2021_27583_MOESM12_ESM.pdf]

nature research

Corresponding author(s): Libusha Kelly & Martin Polz

Last updated by author(s): Sep 16, 2021

Reporting Summary

Nature Research wishes to improve the reproducibility of the work that we publish. This form provides structure for consistency and transparency in reporting. For further information on Nature Research policies, see Authors & Referees and the Editorial Policy Checklist.

Statistics

For all statistical analyses, confirm that the following items are present in the figure legend, table legend, main text, or Methods section.

n/a	Confirmed	
☐	☒	The exact sample size (n) for each experimental group/condition, given as a discrete number and unit of measurement
☒	☐	A statement on whether measurements were taken from distinct samples or whether the same sample was measured repeatedly
☐	☒	The statistical test(s) used AND whether they are one- or two-sided<br>*Only common tests should be described solely by name; describe more complex techniques in the Methods section.*
☐	☒	A description of all covariates tested
☒	☐	A description of any assumptions or corrections, such as tests of normality and adjustment for multiple comparisons
☐	☒	A full description of the statistical parameters including central tendency (e.g. means) or other basic estimates (e.g. regression coefficient) AND variation (e.g. standard deviation) or associated estimates of uncertainty (e.g. confidence intervals)
☐	☒	For null hypothesis testing, the test statistic (e.g. F, t, r) with confidence intervals, effect sizes, degrees of freedom and P value noted<br>*Give P values as exact values whenever suitable.*
☒	☐	For Bayesian analysis, information on the choice of priors and Markov chain Monte Carlo settings
☒	☐	For hierarchical and complex designs, identification of the appropriate level for tests and full reporting of outcomes
☐	☒	Estimates of effect sizes (e.g. Cohen's d, Pearson's r), indicating how they were calculated

Our web collection on statistics for biologists contains articles on many of the points above.

Software and code

Policy information about availability of computer code

Data collection

Data was collected into Excel spreadsheets on a machine with a Windows operating systems.

Data analysis

Bacterial genome tree:
RiboTree (no version ID, https://github.com/philarevalo/RiboTree)

hsp60 trees and visualization:
muscle v.3.8.31
FastTree v.2.1.8
PareTree v.1.0.2
iToL (itol.embl.de)

Viral protein clustering:
MMseqs2 v. 2.23394

Viral protein cluster annotation:
InterProScan v.5.39-77.0
eggNOG-mapper v.2
Meta-iPVP

Functional annotation of viral proteins:
PSI-BLAST (https://blast.ncbi.nlm.nih.gov/Blast.cgi?PAGE_TYPE=BlastSearch&PROGRAM=blastp&BLAST_PROGRAMS=psiBlast)
MPI Bioinformatics implementation of HHpred (no version ID, https://toolkit.tuebingen.mpg.de/tools/hhpred)
EMBL-EBI implementation of JACKHMMER (https://www.ebi.ac.uk/Tools/hmmer/search/jackhmmer)

Annotation of receptor binding proteins:

Phyre2 v.2.0 (http://www.sbg.bio.ic.ac.uk/phyre2/html/page.cgi?id=index)
HHpred

Annotation of recombinases:
InterProScan
CytoScape v.3.3.0
ClusterMaker2 v.1.2.1 Cytoscape plugin

Viral genome clustering:
VICTOR (no version ID, https://ggdc.dsmz.de/victor.php)
OPTSIL (no version ID, within VICTOR at https://ggdc.dsmz.de/victor.php)
VIRIDIC (no version ID, http://rhea.icbm.uni-oldenburg.de/VIRIDIC/)

Viral genome relatedness:
BBtools
vConTACT2 v.0.9.10

Infection matrix structure
BiMat 1.0

Average nucleotide identity of modules
FastANI 1.3

Host range divergence and visualizations:
R 3.6.1 with packages:
ggrepel 0.8.1
ape 5.3
combinat 0.0-8
Infotheo 1.2.0
philentropy 0.4.0.9000
igraph 1.2.4.1
ggraph 2.0.0
cowplot 1.0.0
data.table 1.12.2
ggplot2 3.2.1
tidyverse 1.3.0
ggtree 2.0.4
patchwork 1.1.1

Genome diagrams and visualizations:
R 3.6.1 with packages:
genoplotR 0.8.10
ape 5.4-1
readr 1.3.1

Recombination:
HomBlocks 1.0
progressiveMauve (build 2015/02/13)
trimAI 1.2
IQ-TREE 1.6.12
ClonalFrameML 1.12
Mash 2.2.2
BRIG
MetaCHIP
DWGSIM 1.1.11
FastViromeExplorer

Lysogeny
fastp v.0.20.1
bwa v.0.7.17
samtools v.1.8
bedtools v.2.29.2
bedops v.2.4.39
conda v.4.9.0

General data management
Excel v2108

For manuscripts utilizing custom algorithms or software that are central to the research but not yet described in published literature, software must be made available to editors/reviewers. We strongly encourage code deposition in a community repository (e.g. GitHub). See the Nature Research guidelines for submitting code & software for further information.

Data

Policy information about availability of data

All manuscripts must include a data availability statement. This statement should provide the following information, where applicable:

- Accession codes, unique identifiers, or web links for publicly available datasets
- A list of figures that have associated raw data
- A description of any restrictions on data availability

Data availability
Information about bacteria and phage isolates, infections, and BiMat module assignments are provided in Supplementary Data 1. Summary data describing host taxa by phage taxa and morphotypes are provided in Supplementary Data 2. Information on assignments of phages to VIRIDIC groups are in Supplementary Data 3. Results of vConTACT analyses are available in Supplementary Data 4, this includes a list of phages obtained from the group website of Andrew Millard http://s3.climb.ac.uk/ADM_share/crap/website/26Aug2019_phages.gb.gz. Summary data describing mapping of bacterial genome reads to phage genomes are provided in Supplementary Data 5. Annotations of all proteins and protein clusters of phages in this work are provided in Supplementary Data 6. Information regarding annotation phage recombinases is provided in Supplementary Data 7, this includes a table describing seed sequences based on the original source information at http://biodev.extra.cea.fr/virfam/table.aspx. Source data are provided with this paper in the Supplementary Information/Source Data file for the underlying the matrices in Figure 2, the underlying killing concordance and recombination plots in Figure 4, underlying host and nucleotide sharing in Figure 5, and the cross mapping information in Supplementary Figure 7. New bacterial genomes and hsp60 sequence data deposited with this work are included under the Nahant Collection of NCBI BioProject with accession number PRJNA328102 [https://www.ncbi.nlm.nih.gov/bioproject/?term=PRJNA328102].

Field-specific reporting

Please select the one below that is the best fit for your research. If you are not sure, read the appropriate sections before making your selection.

☐ Life sciences ☐ Behavioural & social sciences ☒ Ecological, evolutionary & environmental sciences

For a reference copy of the document with all sections, see nature.com/documents/nr-reporting-summary-flat.pdf

Ecological, evolutionary & environmental sciences study design

All studies must disclose on these points even when the disclosure is negative.

Study description	Experiments analyzed in this study include a phage predation bait assay and a phage host range assay. For the phage predation assay purified bacterial isolates from 3 days (>1000 isolates) were exposed to viral concentrates collected on the same 3 days and the viral colonies (plaques) enumerated and preserved; new data for this work is reported here. For the host range assay, approximately 250 viruses were assayed for host range on >250 bacterial strains; description of the experimental methods and related data collection for this assay, and a graphical representation of the phage host ranges, were previously presented in (https://doi.org/10.1038/nature25474) where the focus was on one subgroup of viruses; additional data and analyses of the host range assay results are reported here.
Research sample	The research sample in the bait assay was a set of >1000 bacterial strains isolated from water samples collected on three days. This sample was meant to represent bacterial strains predominantly from the family Vibrionaceae, targeted by plating onto selective media, and the rationale for the sampling regime was that targeting a particular family and sampling multiple size fractions over multiple days would allow for representation of both the breadth and depth of strain level diversity within the family. The research sample in the host range assay was a set of approximately 250 viruses and plaque-positive hosts from the bait assay, this sample was meant to represent the populations of phages infecting bacterial strains within the Vibrionaceae, and the rationale was that by selecting one plaque from each bacterial strain showing plaques in the plaque assay it would be possible to obtain a broad overview of the phylogenetic diversity of phages infecting this host group at this site during this time period.
Sampling strategy	The aim of the study was to resolve features of phage predation at the bacterial strain and species levels and to understand these in light of phage genomic diversity, thus the bacterial sample was defined as representatives of the Vibrionaceae that could be isolated on a given day from the coastal ocean and the viruses included were selected such that at least one virus for each plaque-positive bacterial strain was included. Sample sizes met or exceeded those shown in previous studies to be sufficient to reveal strain- and population-level diversity of bacteria (for example, as in Hunt & Lawrence et al. 2008 Science, https://doi.org/10.1126/science.1157890, where approximately 1000 isolates were characterized using the same fractionation, isolation, and partial hsp60 gene sequencing based characterization approaches) and were determined based on logistical constraints of the number of personnel hours required to process samples.
Data collection	Results of the bait assay were recorded by reviewing agar overlay plaque assays throughout the incubation period and counting plaques, KK performed data collection and recording. Results of the host range assay were recorded by reviewing agar overlay host range assay plates throughout the incubation period and marking positive interactions, all results were recorded at the end of the assay based on marks on the plates, results were logged into semi-automated Excel spreadsheets, KK performed data collection and recording.
Timing and spatial scale	Environmental samples from which bacteria and viruses were isolated were collected on three days within the context of a longer time series sampling campaign described elsewhere (https://doi.org/10.1038/s41467-017-02571-4): August 10, 2010; September 18, 2020; and October 13, 2020. These dates were selected to ensure spread across the longer time series sampling and to allow for logistical considerations related to preparation for and processing of new isolates. Samples from each day were collected from three sub-sites at the sampling location each approximately equidistant from shore and reached by wading into the water to a depth of

approximately 1-1.3m and within approximately 40m of each other; on days with excess wave action samples were collected from near the water's edge.

Data exclusions

Sets of phages and bacteria used in the host range matrix analyses are identified in the Methods and in Supplementary Data File 1 together with the criteria defining their assignments to these sets, in brief, these subsets reflect different amounts of data (e.g. hsp60 or genome sequence) available for a particular phage or bacterial strain and whether a given phage is a technical replicate of another based on its deriving from the same plaque in the bait assay. As described in the methods here, and previously (https://doi.org/10.1038/nature25474), in the host range assay: At the termination of the experiment, all positives were called, blind to corresponding replicates, and sizes of clearings at each time point were recorded. Potential for cross-contamination was assessed by visual inspection and considered in final conservative manual curation of 'positive' infection calls. As a result, some cases with 3/3 positive replicates were discarded due to high probability of cross-contamination and some cases with 2/3 positive replicates were included when, for example, these were the only positives on a test plate.

Reproducibility

The bait assays exposing bacterial isolates to lytic phages were performed as single replicates; phage concentrate sample material was limited and the choice was made to commit this material to surveying a large number of independent bacterial isolates for phage predators. General use of virus concentrates generated by the iron chloride/oxalate method in agar overlay assays has shown this method to yield consistent plaque counts and subsequent studies with viral concentrate material from additional days in the time series (not considered in the present manuscript) show smooth shifts in abundance of phage predators on selected strains over time. In the host range assay, as previously reported (https://doi.org/10.1038/nature25474): Each interaction was tested in triplicate, with three separate drop spots arrayed haphazardly into 3 sectors of a 96-well plate in an agar overlay drop spot assay; observations in subsequent smaller-scale host range assays with members of these collections have been consistent with those described here.

Randomization

Bait assays to observe plaque forming phages present in phage concentrates from a given day were performed for a haphazardly but consistently selected subset of bacterial strains isolated for a given day by(selecting the same rows in 96-well cryopreservation plates for each size fraction sampled for each day assayed; there were no other covariates relevant to this experiment to control. Bacterial strains from a given day were assayed with phage concentrates from the same day and associated protocols were performed consistently for bait assays for each of the three separate sampling days. Viruses were selected for purification, genome sequencing, and inclusion in the host range assay haphazardly to represent at least one phage from each plaque-positive host in the assay; as described in the methods here and previously reported (https://doi.org/10.1038/nature25474); the position in each of the three sectors of the 96-well plate arrays used in the host range assay were haphazardly assigned for each of the three viral lysate replicates.

Blinding

Bait assays were not performed blinded, the isolation day, size fractions, and sampling replicates were known for the isolates being assayed as this information was in the sample label but the species identification was not available until later in the study. As previously reported for the host range assay (https://doi.org/10.1038/nature25474): Results of the host range assay were performed blinded insofar as 1) they were recorded without reference to position of the three haphazardly assigned replicates on a given assay plate, and 2) a large number of assays with different sets of viruses were recorded at the same time reducing likelihood of pattern detection.

Did the study involve field work? ☒ Yes ☐ No

Field work, collection and transport

Field conditions

Samples described in this study were collected on three days that were part of a longer time series for which daily conditions were described in detail in Supplementary File 7 of https://doi.org/10.1038/s41467-017-02571-4. In brief, conditions for these three days were as follows. Ordinal day 222: August 10, 2010, air temperature ~25.6°C, water temperature ~13.8°C, clear skies. Ordinal day 261: September 18, 2020, air temperature ~19°C, water temperature ~16.3°C, clear skies. Ordinal day 286: October 13, 2020, air temperature ~13.5°C, water temperature ~14.2°C, clear skies.

Location

The sampling site was Canoe Cove, Nahant, MA, USA (Lat: 42° 25′ 10.6″ N, Lon: 70° 54′ 24.2″ W), samples were collected by wading into the ocean.

Access and import/export

Access to the sampling site was through a public access beach as well as a beach site associated with the Northeastern University Marine Science Center on Nahant. In July 2010, prior to the sampling, permission for access for the sampling period (July - October 2010) was coordinated by Sarah Phenix, Marine Science Laboratory Manager, Northeastern University Marine Science Center, and required institutional releases were obtained.

Disturbance

Water samples were collected by wading into the seawater, with some processing occurring on shore - these procedures did not result in any disturbance nor was anything left at the study site.

Reporting for specific materials, systems and methods

We require information from authors about some types of materials, experimental systems and methods used in many studies. Here, indicate whether each material, system or method listed is relevant to your study. If you are not sure if a list item applies to your research, read the appropriate section before selecting a response.

Materials & experimental systems

n/a	Involved in the study
☒	☐ Antibodies
☒	☐ Eukaryotic cell lines
☒	☐ Palaeontology
☒	☐ Animals and other organisms
☒	☐ Human research participants
☒	☐ Clinical data

Methods

n/a	Involved in the study
☒	☐ ChIP-seq
☒	☐ Flow cytometry
☒	☐ MRI-based neuroimaging
