# Supplementary material for: Resolving the structure of phage–bacteria interactions in the context of natural diversity
Source: Nat Commun. 2022 Jan 18;13:372. doi: 10.1038/s41467-021-27583-z (PMC8766483; doi:10.1038/s41467-021-27583-z)

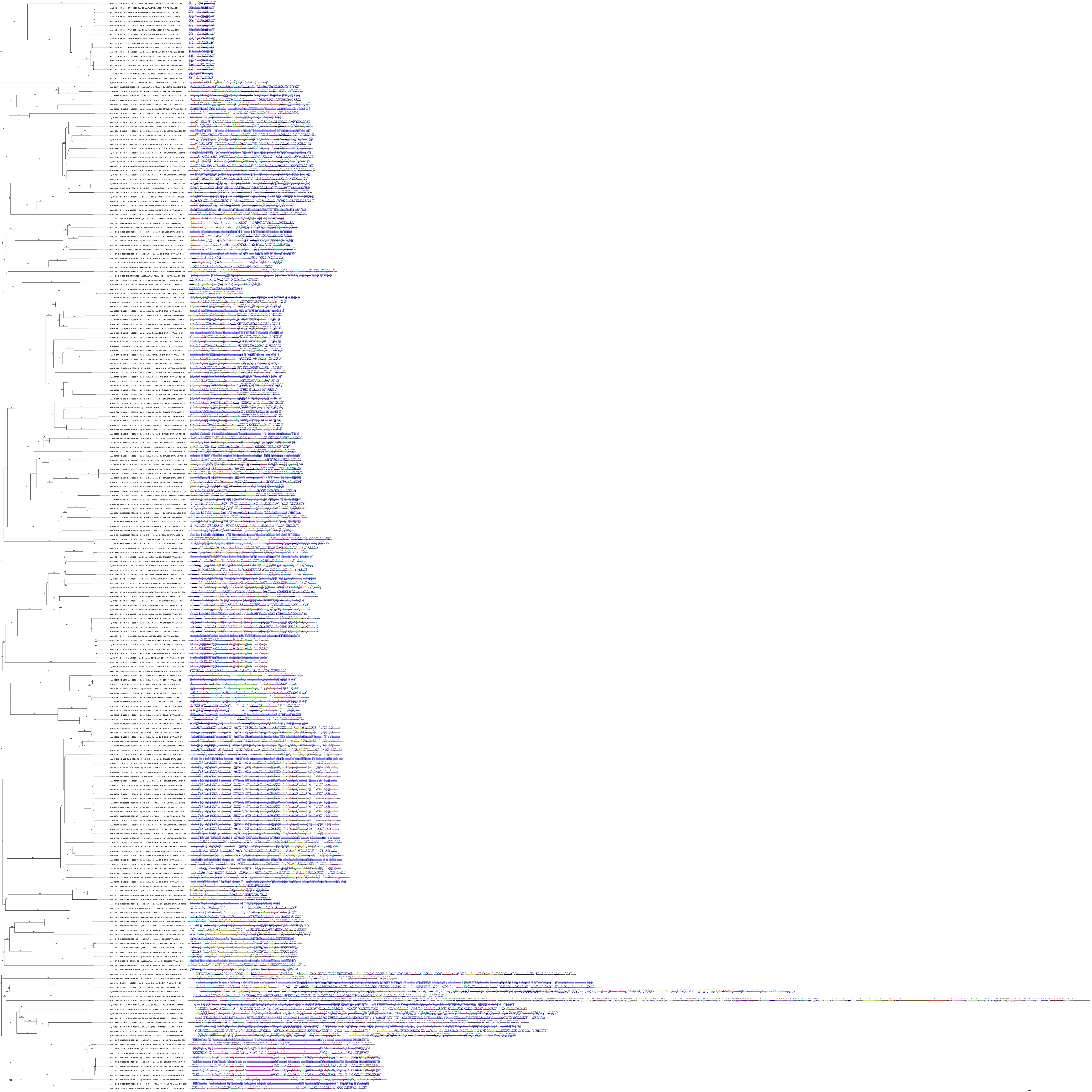

Supplement: Supplementary file 10 — Supplementary Data 8 [file SuppData_8.pdf › SuppData_8.pdf]
