# Supplementary material for: Resolving the structure of phage–bacteria interactions in the context of natural diversity
Source: Nat Commun. 2022 Jan 18;13:372. doi: 10.1038/s41467-021-27583-z (PMC8766483; doi:10.1038/s41467-021-27583-z)

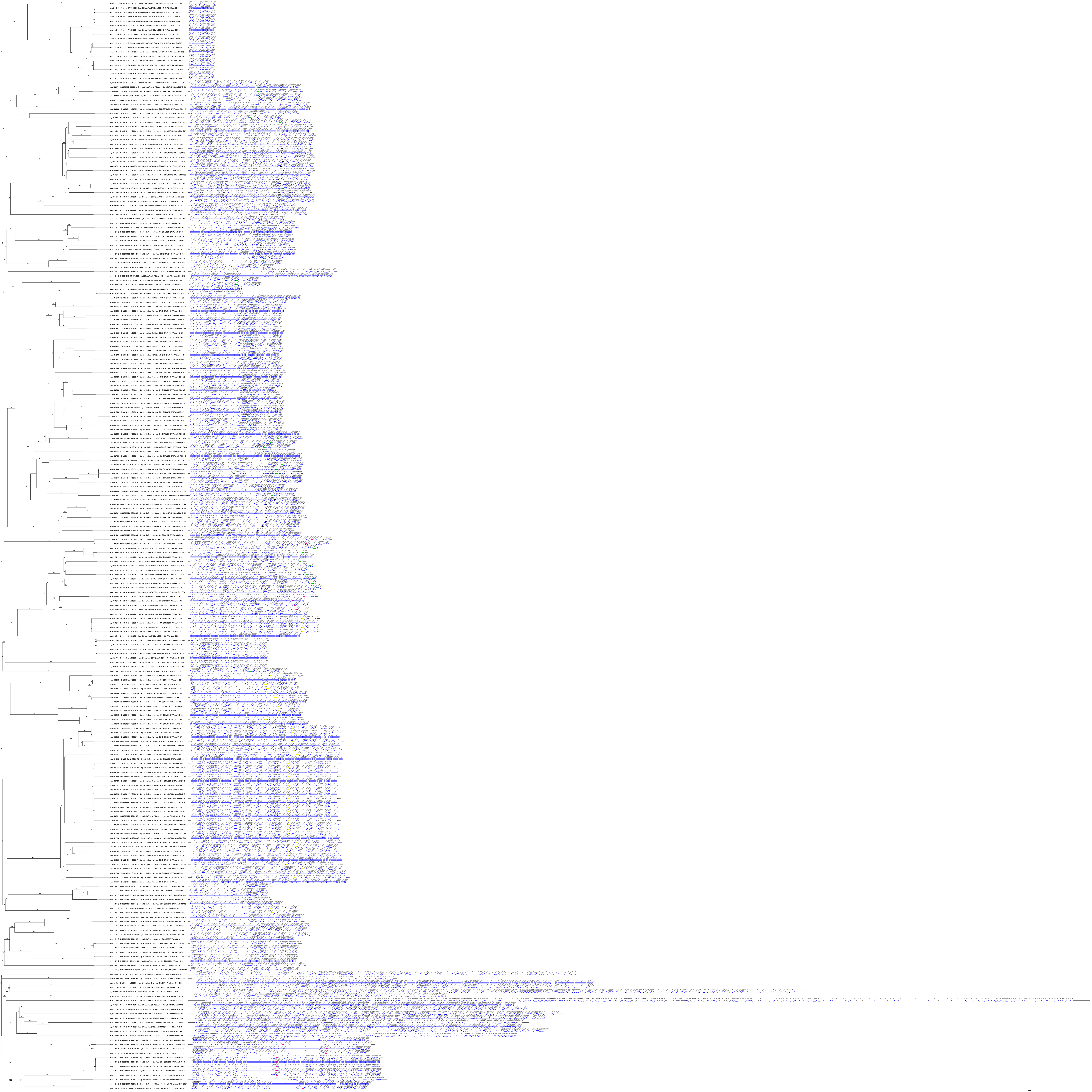

Supplement: Supplementary file 11 — Supplementary Data 9 [file SuppData_9.pdf › SuppData_9.pdf]
